# Chronic effects of different exercise modalities on executive function in Chinese individuals with substance use disorders: a systematic review and network meta-analysis

**DOI:** 10.3389/fpsyg.2026.1979254

**Published:** 2026-10-27

**Authors:** Mingyu Lu, Ying Tian, Jiaxin Wang, Yunqiu Liang

**Affiliations:** College of Sports Science, Shenyang Normal University, Shenyang City, Liaoning, China

**Keywords:** addiction, cognitive flexibility, exercise, inhibitory control, meta-analysis, working memory

## Abstract

**Objective:**

To systematically evaluate the chronic effects of different exercise modalities on executive function in Chinese individuals with substance use disorders (SUD) through a network meta-analysis.

**Methods:**

PubMed, Web of Science, Embase, Cochrane Library, MEDLINE, CNKI, Wanfang Data, and VIP databases were systematically searched from inception to January 2026. The risk of bias of included randomized controlled trials (RCTs) was assessed using the Cochrane ROB 2.0 tool, and the certainty of evidence was evaluated using CINeMA. Statistical analyses were performed using Stata 15.0 and RevMan 5.4.

**Results:**

Traditional pairwise meta-analysis showed that aerobic exercise (AE) significantly improved inhibitory control in Chinese individuals with SUD. Subgroup analysis indicated that, compared with treatment as usual (TAU), a single 30-min session possibly had no significant effect. The network meta-analysis revealed that: for inhibitory control, resistance exercise (RE) potentially ranked highest (SUCRA = 96.7%); for working memory, mind–body exercise (MBE) potentially ranked highest (SUCRA = 99.4%); and for cognitive flexibility, AE potentially ranked highest (SUCRA = 68.3%). Pairwise comparisons suggested that RE was possibly superior to AE in improving inhibitory control, and MBE was possibly superior to RE and AE in improving working memory. The certainty of evidence was mostly low or very low. Importantly, SUCRA ranking estimates should be interpreted with considerable caution.

**Conclusion:**

Different exercise modalities may exert differential effects on specific executive function subdomains in Chinese individuals with SUD. However, given the sparse network structure, clinically fragile transitivity, and low certainty of evidence, all findings should be considered exploratory and hypothesis-generating rather than definitive clinical guidance. Future large-scale, multicenter RCTs are warranted to validate the stability of these results.

**Systematic review registration:**

https://www.crd.york.ac.uk/PROSPERO/view/CRD420261351367, CRD420261351367.

## 1 Introduction

Substance Use Disorders (SUD) is a treatable, chronic medical condition defined by a problematic pattern of substance use leading to clinically significant impairment or distress, encompassing cognitive, behavioral, and physiological symptoms that reflect continued use despite harm, and ranging from mild to severe, with severe forms commonly referred to as addiction (Volkow et al., 2016). According to the United Nations Office on Drugs and Crime (UNODC), the global number of people who used drugs rose to 316 million in 2023, representing a 28% increase over the past decade and accounting for 6% of the global adult population; cannabis, opioids, amphetamines, cocaine, and ecstasy remain the most commonly used substances worldwide, the expanding synthetic drug market, driven by low production costs and reduced risks of detection, has led to record highs in cocaine production and amphetamine-type stimulant seizures; SUD imposes substantial health and societal costs, with an estimated 463,000 deaths and 27.7 million healthy life years lost due to disability and premature mortality; notably, only one in twelve individuals with drug use disorders received any form of treatment in 2023, highlighting a significant global treatment gap (UNODC, 2025). Individuals with SUD often exhibit clinical symptoms such as hypertension, syncope, tachycardia, epilepsy, anxiety, and depression (Koob and Volkow, 2010). Executive function is a crucial higher-level neural processing process within the human brain’s cognitive system. For individuals with SUD, executive function is a key target for resisting the temptation of drug cues and achieving successful withdrawal, as well as a core predictor of relapse. Long-term drug use can cause irreversible damage to the brain’s executive function, manifesting as reduced activity in the prefrontal cortex, dysfunction of the dopamine system mediated by the dysregulation of the fronto-striatal neural circuit. This leads to weakened inhibitory control, decline in working memory, increased decision-making impulsivity, difficulty in flexible behavioral strategy switching, making it challenging to suppress automatic psychological craving for drugs and to effectively evaluate the trade-offs between delayed rewards and immediate gratification, thus falling into a solidified pattern of drug use behavior. This further exacerbates compulsive drug-seeking behavior, forming a vicious cycle and increasing the probability of relapse (Potenza, 2014; Ceceli et al., 2023).

Currently, the mainstream treatment for SUD remains pharmacotherapy and psychosocial rehabilitation. Although receptor agonist substitution can effectively alleviate withdrawal symptoms, the substitution drugs themselves carry risks of abuse and physiological dependence, potentially leading to secondary addiction (Yates, 2024); while psychological therapies such as group counseling and cognitive behavioral therapy are widely adopted, they face challenges such as a shortage of therapists, poor patient compliance, high costs, and high dropout rates in long-term follow-up, making it difficult to achieve sustainable symptom management and behavioral reshaping (Bahji, 2025). The World Health Organization (WHO) points out that exercise, as a low-cost, non-pharmacological, and adjunctive intervention, is widely used in drug rehabilitation training. By increasing the body’s dopamine concentration and binding to dopamine receptors, it activates the same reward pathways as abused drugs, which can effectively reduce the relapse rate among individuals with SUD and improve comorbid mental health problems (World Health Organization, 2019; Volkow et al., 2004). Its efficacy has shown good persistence and stability in long-term follow-up. A growing body of evidence suggests that exercise positively impacts multiple EF subdomains by promoting the secretion of Brain-Derived Neurotrophic Factor (BDNF), enhancing neuroplasticity, and improving cerebral blood flow (Cefis et al., 2023).

Currently, there are numerous types of exercise-based drug rehabilitation methods. Selecting the optimal exercise modality can more efficiently achieve targeted recovery of executive function impairments associated with dopamine. Research indicates that AE, RE, MBE, and HIIT can all significantly improve executive function in individuals with SUD. For example, Tai Chi, characterized as a MBE integrating physical movements with mental concentration, controlled respiration, and relaxation, has been shown to exert significant beneficial effects on executive function in methamphetamine-dependent individuals (Menglu et al., 2021). However, current research findings still have several limitations. First, at the individual level, the efficacy of exercise interventions is modulated by factors including age, sex, type of substance abused, and duration of addiction, resulting in marked inter-individual variability in treatment outcomes. Such variability complicates the extrapolation of findings from single studies to the broader addicted population. Second, at the level of intervention protocols, there is substantial heterogeneity across studies in terms of exercise type, intensity, frequency, and session duration. Moreover, most studies have reported only global improvements in executive function, whereas specific exercise regimens optimized for its subdomains remain scarce. More critically, the existing evidence is overwhelmingly derived from trials comparing a single exercise modality with TAU. Consequently, clinicians are unable to determine the comparative effectiveness of different exercise modalities for improving executive function based on current evidence when multiple options are available.

Therefore, we conducted a network meta-analysis integrating direct and indirect evidence to evaluate and rank multiple exercise interventions, and performed subgroup analyses to examine dose–response relationships, thereby offering preliminary exploratory evidence for patient health promotion and rehabilitation.

## 2 Methods

### 2.1 Literature search strategy

This study strictly followed the PRISMA guidelines (Page et al., 2020), and two researchers independently and blindly searched PubMed, Embase, Web of Science, Cochrane Library, MEDLINE, CNKI, Wanfang Data, and VIP databases. The search period was from the establishment of the databases to January 2026, with language restrictions limited to Chinese and English. Potential studies were retrieved by combining the following terms: Chinese search terms: exercise, mind–body exercise, aerobic exercise, resistance exercise, high-intensity interval training, drug addiction, heroin, methamphetamine, executive function, inhibitory control, cognitive flexibility, working memory, randomized controlled trials, etc.; English terms: exercise, mind–body exercise, aerobic exercise, resistance exercise, high-intensity interval training, amphetamine, heroin, substance use disorders, drug dependence, executive function, RCT, etc. Subject terms were combined using the Boolean logical operator “AND” for retrieval, and comprehensiveness was ensured by manually searching the references and systematic review studies. Taking PubMed as an example for English databases, the search strategy is shown in Table 1.

**Table 1:** Search strategy for PubMed.

Step	Retrieval
#1	((((((((((((((((((((((((((((Exercise[MeSH Terms]) OR (Exercise, Physical[Title/Abstract])) OR (Physical Exercise[Title/Abstract])) OR (Exercise, Isometric[Title/Abstract])) OR (Isometric Exercise[Title/Abstract])) OR (Aerobic Exercise[Title/Abstract])) OR (Exercise, Aerobic[Title/Abstract])) OR (Exercise Training[Title/Abstract])) OR (Training, Exercise[Title/Abstract])) OR (Physical Activity[Title/Abstract])) OR (Activities, Physical[Title/Abstract])) OR (Activity, Physical[Title/Abstract])) OR (Physical Activities[Title/Abstract])) OR (Active Breaks[Title/Abstract])) OR (Activity Breaks[Title/Abstract])) OR (Acute Exercise[Title/Abstract])) OR (Exercise, Acute[Title/Abstract])) OR (Training, Resistance[Title/Abstract])) OR (Resistance train[Title/Abstract])) OR (Resistance exercise[Title/Abstract])) OR (High Intensity Interval Training[Title/Abstract])) OR (HIIT[Title/Abstract])) OR (High-Intensity Interval Training[Title/Abstract])) OR (Mind - body exercise[Title/Abstract])) OR (Chinese exercise[Title/Abstract])) OR (traditional exercise[Title/Abstract])) OR (Chinese traditional exercise[Title/Abstract])) OR (traditional exercise[Title/Abstract])) OR (Traditional Chinese Exercise[Title/Abstract])
#2	((((((((((((((((((((Substance-Related Disorders[MeSH Terms]) OR (Disorders, Substance Related[Title/Abstract])) OR (Related Disorders, Substance[Title/Abstract])) OR (Drug Habituation[Title/Abstract])) OR (Drug Use Disorders[Title/Abstract])) OR (Substance Abuse[Title/Abstract])) OR (Substance Dependence[Title/Abstract])) OR (Chemical Dependence[Title/Abstract])) OR (Substance Addiction[Title/Abstract])) OR (Substance Use Disorders[Title/Abstract])) OR (Drug Dependence[Title/Abstract])) OR (Drug Addiction[Title/Abstract])) OR (Drug Abuse, Prescription[Title/Abstract])) OR (Drug Abuse[Title/Abstract])) OR (Amphetamine[Title/Abstract])) OR (Cocaine[Title/Abstract])) OR (Heroin[Title/Abstract])) OR (Methadone[Title/Abstract])) OR (Morphine[Title/Abstract])) OR (Opioid[Title/Abstract])) OR (Cannabis[Title/Abstract])
#3	(((((Executive Function[MeSH Terms]) OR (Function, Executive[Title/Abstract])) OR (Executive Control[Title/Abstract])) OR (Inhibitory control[Title/Abstract])) OR (Working memory[Title/Abstract])) OR (Cognitive flexibility[Title/Abstract])
#4	#1 AND #2 AND #3

### 2.2 Inclusion and exclusion criteria

Develop research inclusion and exclusion criteria based on the PICOS principles (Munn et al., 2018). Inclusion criteria: (1) Study subjects are individuals with SUD, meeting the diagnostic criteria for SUD in the Diagnostic and Statistical Manual of Mental Disorders (5th ed.) (Battle, 2013), it should be noted that, although SUD encompasses a broad range of substances, including alcohol and tobacco, according to the DSM-5 criteria, the present study focused on illicit drugs (include methamphetamine, heroin, opioids, cannabis, cocaine, and morphine), regardless of sex or race; (2) Study type is RCT; (3) Intervention is exercise therapy, including aerobic exercise, resistance exercise, mind–body exercise (e.g., Tai Chi, and Qigong), with the control group receiving treatment as usual (including drug prevention education, legal education, daily observation, discussions on drug-related harms, reading, literary and calligraphy practice, and hygiene training; participants were also required to avoid structured physical activity during the observation period) (Wang et al., 2014); (4) The study should adopt a chronic exercise approach for the intervention. (5) Each study reports at least one outcome measure (inhibitory control, working memory, cognitive flexibility), where inhibitory control evaluation tools are Stroop, GO/NO-GO, Flanker, or Stop-signal tasks, working memory uses N-back tasks, and cognitive flexibility uses task-switching tasks. In this meta-analysis, reaction time (RT) was selected as the primary outcome measure for executive function. RT is widely recognized as one of the most sensitive and frequently reported indicators in executive function research (Albinet et al., 2012). In numerous classic executive function paradigms, core metrics are typically derived based on RT (Miyake et al., 2000). Previous meta-analyses have also demonstrated that the beneficial effects of exercise interventions on RT are generally more consistent and pronounced than those on accuracy (Chang et al., 2012). Given that all studies included in this review reported RT, whereas some did not report accuracy, we prioritized the extraction of RT to maintain outcome homogeneity and comparability across studies. For RT outcomes, shorter latencies were coded as beneficial changes (i.e., negative SMD values), as shorter RT reflect superior executive function.

Exclusion criteria: (1) Participants are addicted to substances such as tobacco and alcohol. (2) Duplicate publications. (3) Participants have undergone psychiatric treatment or suffer from other severe illnesses. (4) Participants receive other exercise interventions. (5) Study indicators are not quantified or data are incomplete. (6) Study types are conference abstracts, animal studies, review articles, editorials, and non-English, non-Chinese articles.

### 2.3 Literature screening and data extraction

This study was independently conducted by two researchers strictly adhering to pre-established inclusion and exclusion criteria (Figure 1). Data extraction content (Table 2) included the first author’s name, publication year, study sample size and age, intervention measures, cycle, frequency, single intervention time, outcome indicators, and bias judgment criteria for literature quality assessment. Discrepancies were resolved through discussion with a third researcher.

**Figure 1:**
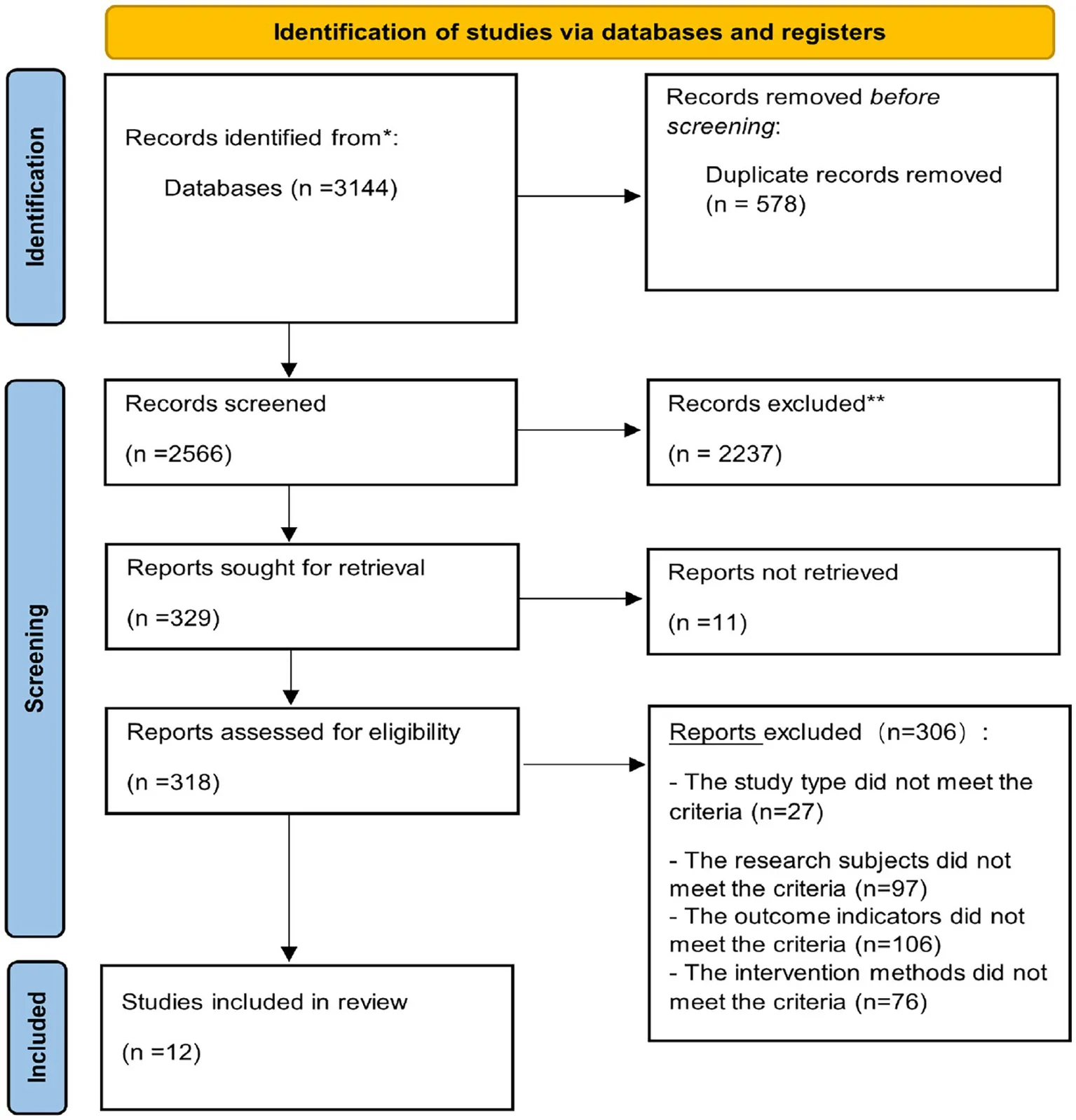
PRISMA flowchart for study selection.

**Table 2:** Characteristics of included studies.

Study	Country	Types of substance (gender)	Sample size (Age)	Types of intervention	Time/frequency	Cycle	Outcome
				Exp/Con			
He (2022)	China	Not reported (Male)	Exp18(31.8 ± 4.7) Con20(34.45 ± 3.9)	②/①	60 min/5 times/week	4 months	①②③
Liu (2021)	China	MA (Male)	Exp42(27.1 ± 4.51) Con39(27.86 ± 4.37)	②/①	60 min/5 times/week	8 weeks	①②③
Zhang et al. (2024)	China	MA (Female)	Exp25(28.31 ± 1.81) Con22(27.28 ± 1.62)	④/①	40 min/2times/week	10 weeks	①
Fang and Zheng (2024)	China	Heroin (Male)	All77 (38.07 ± 7.04)	②③/①	60 min/5times/week	3 months	①②③
Chen et al. (2021)	China	MA (Male)	Exp19(29.58 ± 1.01) Exp17(32.53 ± 1.16) Con21(33.24 ± 1.73)	②/①	30 min/3times/week	8 weeks	①
Menglu et al. (2021)	China	MA (Female)	Exp35(39.31 ± 10.33) Con37(39.37 ± 9.28)	④/②	40 min/3times/week	12 weeks	①②③
Wang et al. (2017)	China	MA	Exp25(32.2 ± 6.97) Con25(34.76 ± 7.96)	②/①	30 min/3times/week	12 weeks	①
Zhu et al. (2023)	China	MA (Male)	Exp32(30.3 ± 5.28) Con32(29.8 ± 5.51)	②/①	60 min/3times/week	8 weeks	①
Zhu et al. (2022)	China	MA (Male)	Exp40(34.61 ± 5.12) Con37(34.94 ± 6.58)	②/①	35 min/5times/week	3 months	①
Liu and Wang (2023)	China	MA (Male)	Exp23(28.48 ± 3.3) Con23(27.66 ± 3.66)	②/①	60 min/5times/week	8 weeks	①②③
He et al. (2021)	China	MA (Male)	Exp16(41.88 ± 13.44) Con16(42.81 ± 11.54)	④/②	25 min/3times/week	4 weeks	①
Xu (2025)	China	Not reported (Male)	Exp12(36.5 ± 5.79) Exp12(36.67 ± 5.84) Con11(38.82 ± 4.64)	④②/①	60 min/3times/week	12 weeks	①②

Methamphetamine: MA; All, Total number of participants; Exp, Experiment Group; Con, Control Group; Types of intervention: ① TAU; ② AE; ③ RE; ④ MBE; Outcome: ① Inhibitory control; ② Working memory; ③ Cognitive flexibility.

### 2.4 Risk of bias assessment

This study employed the latest version of the Cochrane Risk of Bias assessment tool, RoB 2.0, to evaluate the risk of bias in the included studies (Sterne et al., 2019). The unique advantage of this tool lies in its integration of two assessment methods, enabling a more comprehensive evaluation of the risk or degree of bias in intervention measures. The tool categorizes the risk of bias in RCTs into three levels: “low risk,” “some concerns,” or “high risk.” Two researchers independently assessed the risk of bias for the included studies, and any disagreements were resolved through discussion by a third researcher.

### 2.5 Statistical analysis

All outcome variables in this study were continuous, and the effect size for each outcome was expressed as standardized mean difference (SMD) with its 95% confidence interval (95% CI). Prior to pooling effect sizes, the direction of effects was harmonized across all studies. For each eligible study, the means and standard deviations (SDs) of baseline and immediate outcome variables were extracted for both the experimental and control groups. These values were then converted into pre-to-post intervention mean changes and their corresponding standard deviations. The following formulas were used for estimation:


$$\begin{array}{l} \text{mean}\;\Delta \text{Mean}=\text{Meanpost}-\text{Meanpre};\\ \text{standard deviation}\;\mathrm{\Delta SD}=\sqrt{\begin{array}{l} SD_{\text{post}}^{2}+SD_{\mathrm{pre}}^{2}\\ -2*R*SD_{\text{post}}\\ {}*SD_{\mathrm{Pre}} \end{array}}\;\\ \;(R=0.5) \end{array}$$

Statistical analyses were performed using Stata 15.0 and RevMan 5.4. For traditional pairwise meta-analyses, a random-effects model was used for pooling. The magnitude of the effect was classified as: (1) small (g < 0.20), (2) small to medium (g = 0.20–0.49), (3) medium (g = 0.50–0.79), and (4) large (g ≥ 0.80). Heterogeneity was assessed using Cochran’s Q test and the I^2^ statistic. Between-study heterogeneity was examined utilizing the I^2^ statistic, with values ranging from 1–49%, 50–74%, and 75–100% corresponding to low, moderate, and high heterogeneity, respectively. For pairwise meta-analyses, following the approach recommended by the Cochrane Handbook, when a multi-arm trial contributed to a specific pairwise comparison and the experimental intervention arms could not be pooled for clinical reasons, the sample size of the shared control group was divided evenly among the intervention arms being compared. To explore the impact of potential effect modifiers on the pooled estimates, subgroup analyses were conducted, and between-subgroup differences were evaluated using Q test and I^2^ statistic. Publication bias was assessed using Egger’s test and funnel plots (Cumpston et al., 2019).

For network meta-analysis, the analysis was conducted within a frequentist framework. First, a network evidence plot was generated, in which nodes represent interventions (with node size reflecting the sample size of each intervention) and the thickness of edges indicates the number of studies available for each comparison. For multi-arm trials, all intervention arms were retained and included in the network structure, and all possible pairwise comparisons were extracted. To avoid double-counting of the shared control group, the correlations between effect sizes arising from the same study were accounted for by constructing a covariance matrix. Interventions were defined as network nodes, and different forms of the same exercise modality were collapsed into a single node. Global inconsistency was assessed using the design-by-treatment interaction model, and local inconsistency was evaluated using the node-splitting approach. The loop inconsistency test was evaluated using the inconsistency factor (IF) and *p*-value. If the IF is equal to or close to 0, and *p* > 0.05, it indicates that no statistically significant inconsistency was detected within the loops in the current dataset, where IF = |dAB direct − dAB indirect| (AB represents the comparison between two different interventions). In a sparse network, statistical power is limited; non-significant results should not be interpreted as confirming consistency. They merely indicate that no evidence of inconsistency was detected with the available data. The surface under the cumulative ranking curve (SUCRA) was calculated to rank the comparative effectiveness of the interventions (with higher SUCRA values indicating better efficacy). It should be emphasized that SUCRA reflects relative ranking probability rather than absolute treatment magnitude or clinical relevance. High SUCRA values do not necessarily correspond to statistically significant differences between interventions, especially in sparse networks. Finally, comparison-adjusted funnel plots were used to comprehensively evaluate small-study effects and publication bias (Edwards et al., 2023).

## 3 Results

### 3.1 Study selection

A total of 3,144 relevant studies were obtained through preliminary database screening and manual retrieval: CNKI (*n* = 67), Wanfang Data (*n* = 47), VIP (*n* = 18), Cochrane Library (*n* = 422), PubMed (*n* = 764), Embase (*n* = 359), Web of Science (*n* = 935), MEDLINE (*n* = 532). After deduplication using EndNote X9, 2,566 articles remained; after further screening by reading titles and abstracts and excluding irrelevant studies, 318 articles remained; finally, the full texts of the literature were read, and after screening according to the inclusion, exclusion, and literature quality evaluation criteria, 12 studies were finally included.

### 3.2 Characteristics of included studies

A total of 12 RCTs met the inclusion criteria and were included in the qualitative synthesis and network meta-analysis. Among these, 9 were two-arm studies and 3 were three-arm studies, involving a total of 676 individuals with SUD. Four exercise modalities were included. All studies were conducted in China, with publication years ranging from 2017 to 2025. Included studies comprised 9 peer-reviewed journal articles and 3 postgraduate dissertations. Regarding the study population, all participants were diagnosed with SUD. Specifically, the majority of the studies (9 studies) focused on MA dependence, one study focused on heroin dependence, and two studies did not explicitly specify the primary substance of abuse. The duration of exercise interventions ranged from 4 weeks to 4 months. The intervention frequency varied from 2 to 5 sessions per week, with each session typically lasting between 25 and 60 min. After the exercise intervention, tasks were used as a unique method for measuring outcome indicators. During the literature search, one article on HIIT was identified but excluded because it was an acute study. The basic characteristics of the included studies are shown in Table 2.

### 3.3 Risk of bias assessment results

According to the RoB 2.0 assessment results, five studies were rated as high risk of bias (Figure 2). Among these, the domain of “bias due to selection of reported results” had the lowest risk, while the domain of “bias due to deviation from intended interventions” had the highest risk score. Due to the reliance on professional on-site instruction and supervision for the implementation of exercise interventions, the feasibility of blinding participants and intervention implementers is low, thus this domain presents a significant risk of substantial bias.

**Figure 2:**
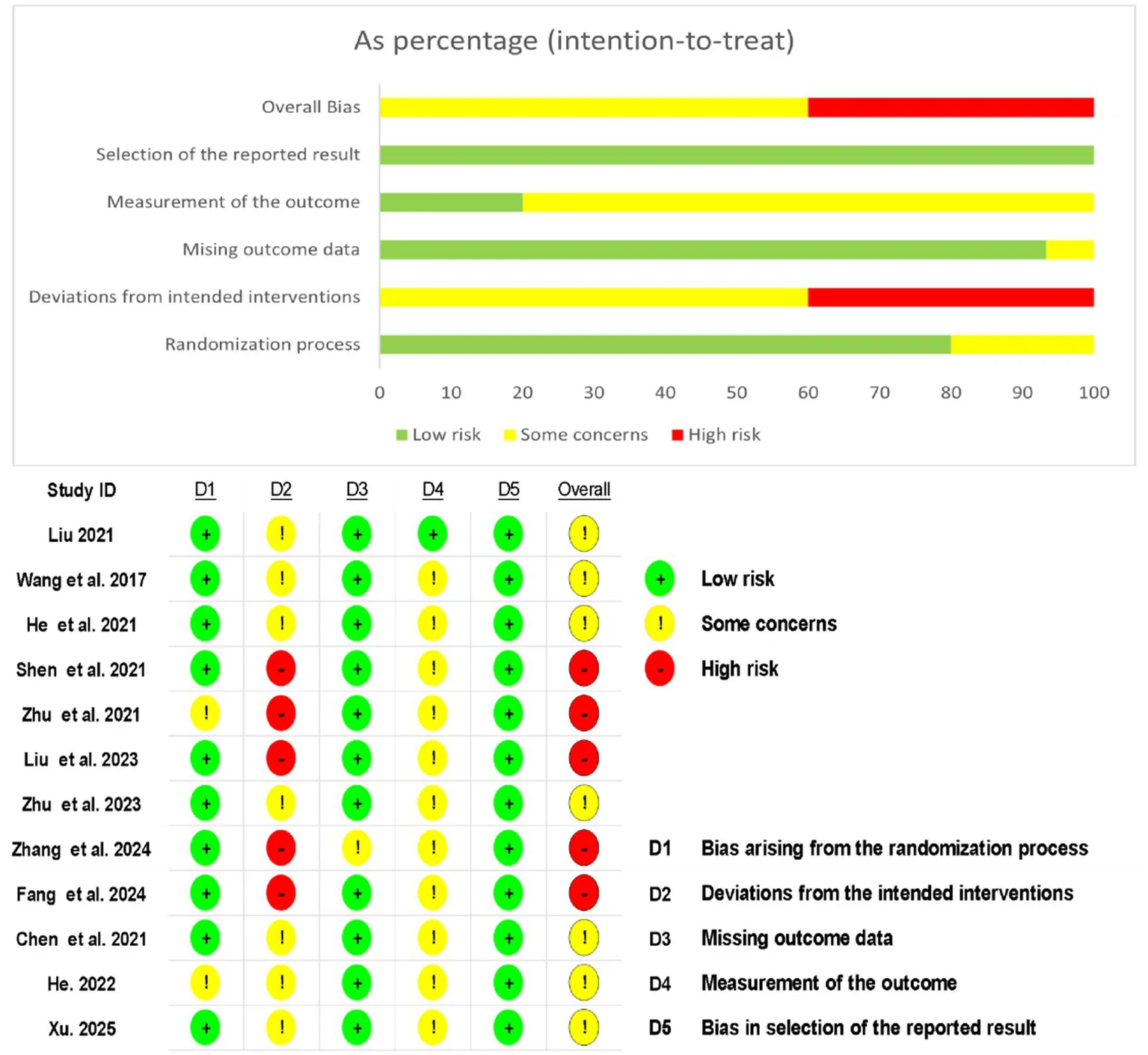
Cochrane RoB 2.0 risk assessment summary.

### 3.4 Results of traditional meta-analysis

Given the number of included studies, both an overall meta-analysis and subgroup analyses were conducted to examine the effects of AE on inhibitory control in Chinese individuals with SUD. Using a random-effects model, compared with TAU, AE [SMD = −0.70, 95% CI (−0.94, −0.47), *p* < 0.001] may significantly improve inhibitory control in individuals with SUD, with studies showing low to moderate statistical heterogeneity (I^2^ = 33%, *p* = 0.15). Publication bias was not detected, as indicated by Egger’s test (*p* = 0.42) and funnel plot asymmetry.

Subgroup analyses were performed on potential effect modifiers to preliminarily explore dose–response relationships (Figure 3). Due to the lack of additional variables such as years of substance use and exercise intensity in the extracted data, the analyses focused on age, session duration, exercise frequency, intervention duration, and assessment tool. Dose variables were defined as follows: age (20–30, 30–40 years), session duration (30, 35–40, 60 min), exercise frequency (1–3, 4–5 times/week), intervention duration (8 or 12 weeks), and assessment tool (Go/No-Go, Flanker, Stop-signal, Stroop). These cut-offs were determined by the distribution of characteristics in the included studies and represent descriptive categorisations rather than *a priori* hypothesis tests. Subgroup analyses revealed that, compared with the control group, a 30-min session of AE [SMD = −0.57, 95% CI (−1.14, 0.01), *p* = 0.05] did not observe a significant improvement, and the interaction effects between all subgroup comparisons were non-significant. (I^2^ = 0, *p* > 0.05). It should be noted that the subgroup analyses examining optimal exercise prescription parameters were based on a limited number of studies within each subgroup. In some cases, a subgroup contained only a single study. Consequently, these analyses should be interpreted as exploratory rather than confirmatory, and the findings should not be used to formulate definitive practical recommendations.

**Figure 3:**
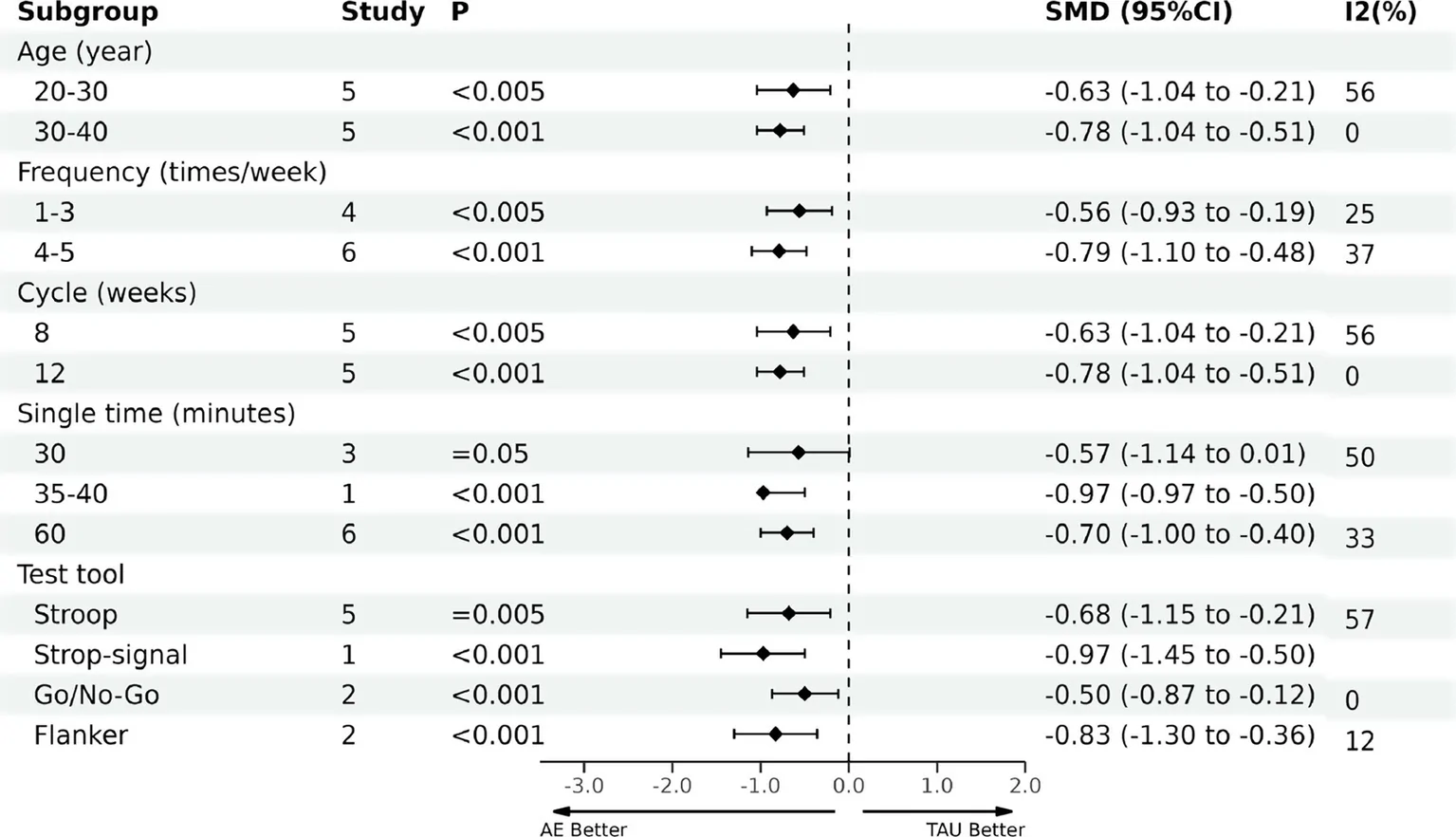
Forest plot of subgroup analyses comparing the effects of AE versus TAU on inhibitory control in individuals with SUD. The left column displays the predefined subgroup variables. The “Study” column indicates the number of included studies per subgroup, and the “P” column represents the statistical significance of the pooled effect within each subgroup. The right column lists the pooled SMD and 95% CI. Diamonds represent point estimates, horizontal lines represent 95% CI, and the vertical dashed line indicates the line of no effect. *p* < 0.05 indicates statistical significance.

Galbraith plots were applied to visualise outliers contributing to heterogeneity in the meta-analysis. As shown in the figure, the X-axis represents precision (x = 1/SE) and the Y-axis represents the standardised individual effect size (y = ES/SE). The diagonal regression line originates from the coordinate system’s origin (0, 0), and its slope represents the pooled overall effect size (Anzures-Cabrera and Higgins, 2010). The two parallel dashed lines (or shaded area) around the regression line denote the approximate 95% confidence intervals, with a width of ±2 units. When the study by Liu and Wang (2023). (Liu and Wang, 2023) was omitted, all remaining studies fell within the 95% confidence interval, suggesting that this study may be the main outlier source of overall heterogeneity (Figure 4).

**Figure 4:**
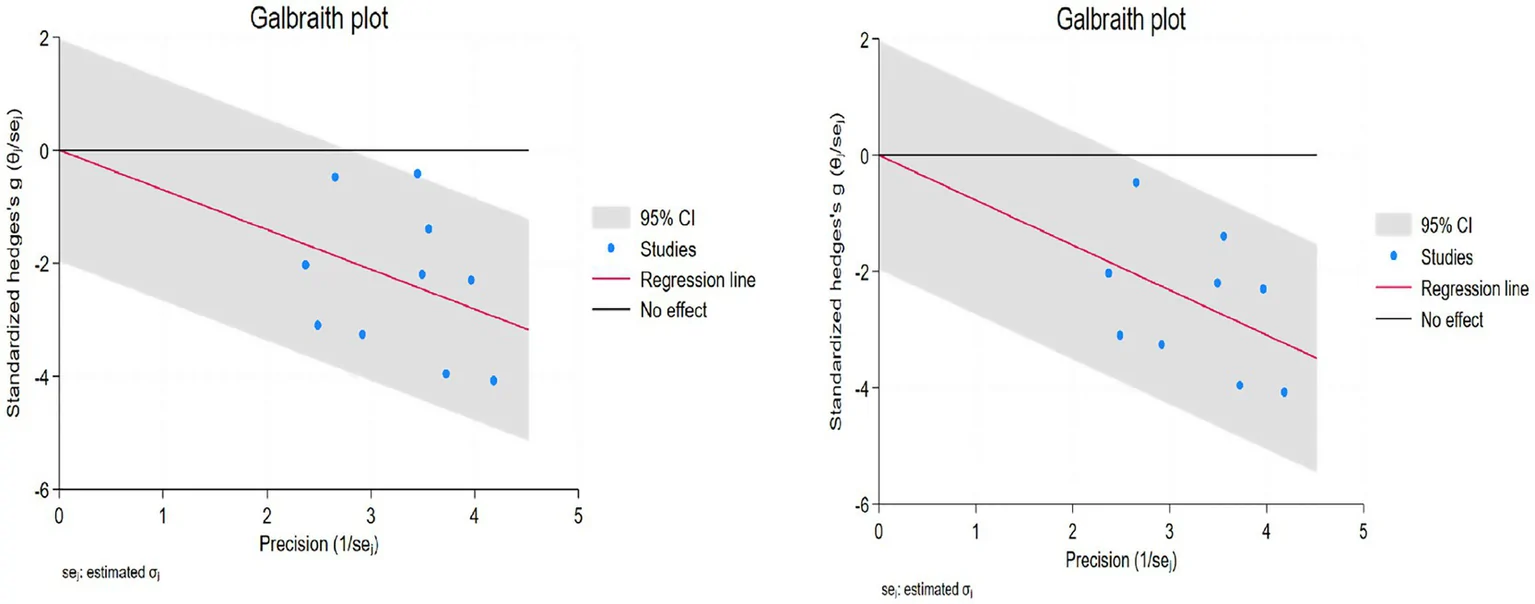
Galbraith plot for evaluating heterogeneity in the effects of AE on inhibitory control in individuals with SUD. Blue dots represent individual studies; the red line indicates the weighted regression line; and the grey shaded area represents the 95% CI.

To assess the robustness of the meta-analytic results, sensitivity analyses were conducted using the leave-one-out method and model transformation (Figure 5). The results indicated that after sequentially omitting each single study, the pooled effect size did not change substantially and the direction of effect remained stable. Liu and Wang (2023) was excluded, the I^2^ value decreased from 33 to 11%, indicating a marked reduction in heterogeneity. This may be attributable to the fact that the AE intervention in that study was performed on an indoor treadmill in a relatively enclosed environment. After the exclusion, the pooled effect [SMD = −0.77, 95% CI (−0.99, −0.56), *p* < 0.001] became more stable, suggesting that the results are more reliable. Overall, the findings indicate that exercise may enhance inhibitory control in Chinese individuals with SUD compared with the control group.

**Figure 5:**
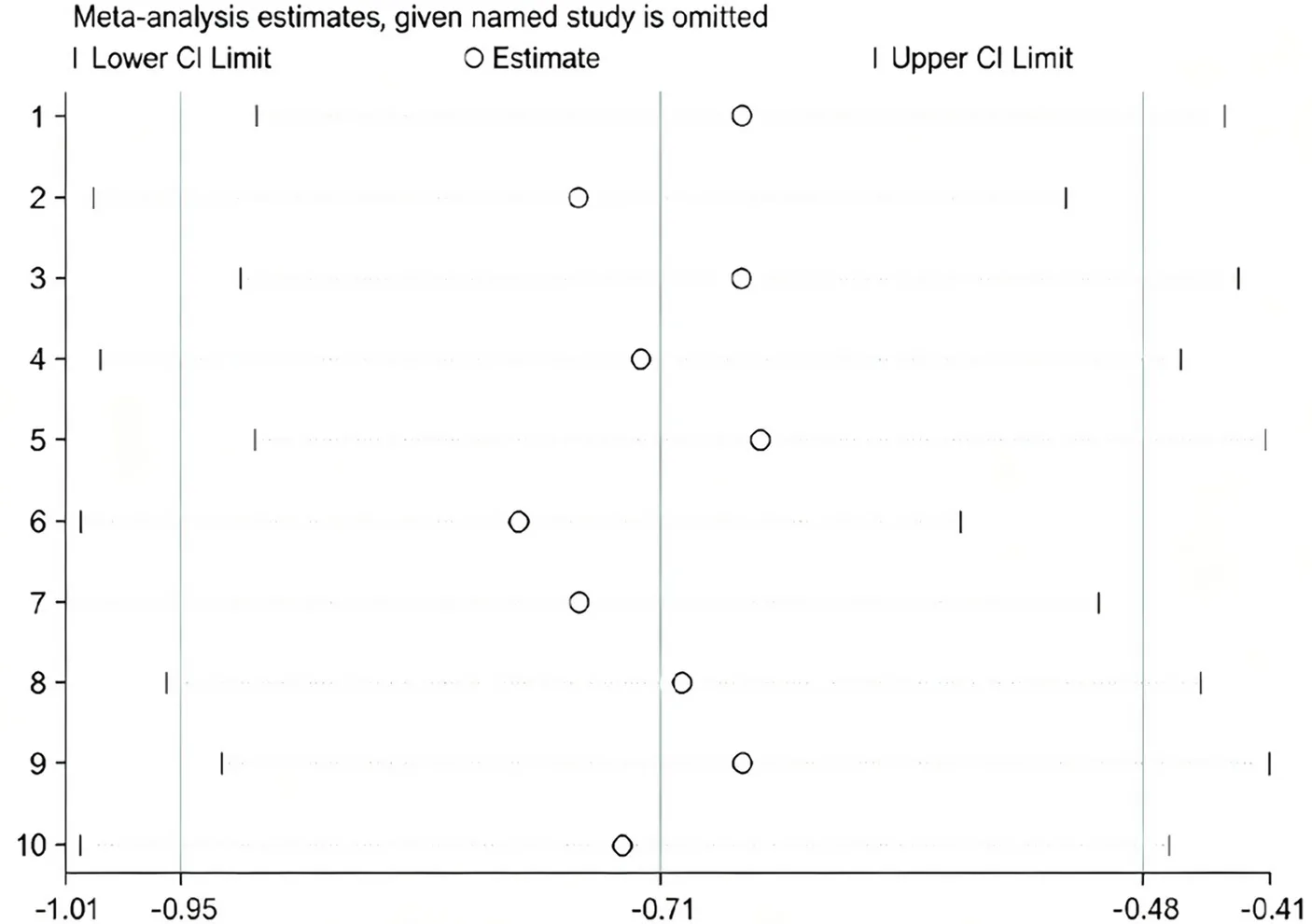
Leave-one-out sensitivity analysis plot. The y-axis lists the sequentially omitted studies (1–10); the x-axis represents the pooled effect sizes of the remaining studies after omitting each study, and the horizontal lines indicate their corresponding 95% CI.

### 3.5 Results of network meta-analysis

#### 3.5.1 Inconsistency test

To assess the reliability of the network meta-analysis results, a global inconsistency test was first performed. The overall consistency test revealed no significant inconsistency for inhibitory control (*p* = 0.28), working memory (*p* = 0.89), and cognitive flexibility (*p* = 0.97), all of which were greater than 0.05, suggesting good overall consistency. Furthermore, the loop inconsistency test results showed that all triangular closed-loop inconsistencies within each outcome were not significant (IF ≈ 0, *p* > 0.05), exhibiting good local consistency. Subsequently, the node-splitting method was employed to evaluate the consistency between local direct and indirect evidence within the executive function subdomain networks. The specific results were as follows: In the inhibitory control network, no significant differences were found between direct and indirect evidence for any pairwise comparison. Specifically, AE versus RE showed *p* = 0.542 (Δ_direct-indirect = −0.176, SE = 0.489), AE versus MBE showed *p* = 0.957 (Δ_direct-indirect = −0.048, SE = 0.881), AE versus TAU showed *p* = 0.971 (Δ_direct-indirect = −0.019, SE = 0.529), RE versus TAU showed *p* = 0.572 (Δ_direct-indirect = −0.264, SE = 0.466), and MBE versus TAU showed *p* = 0.572 (Δ_direct-indirect = −0.049, SE = 0.881), with all test *p* values > 0.05. In the working memory network, AE versus RE showed *p* = 0.770 (Δ_direct-indirect = 0.266, SE = 0.908), AE versus MBE showed *p* = 0.905 (Δ_direct-indirect = −0.084, SE = 0.710), AE versus TAU showed *p* = 0.601 (Δ_direct-indirect = −0.555, SE = 1.062), RE versus TAU showed *p* = 0.916 (Δ_direct-indirect = −0.066, SE = 0.622), and MBE versus TAU showed *p* = 0.905 (Δ_direct-indirect = −0.084, SE = 0.710), with all test *p* values > 0.05. In the cognitive flexibility network, AE versus MBE showed *p* = 0.974 (Δ_direct-indirect = 0.061, SE = 1.865), and MBE versus TAU showed *p* = 0.974 (Δ_direct-indirect = 0.061, SE = 1.865), with all test *p* values > 0.05.

Comprehensive evaluation indicated that, among all estimable comparisons, the confidence intervals for the differences between direct and indirect evidence all included zero, which to some extent supports the use of a consistency model in the subsequent network meta-analysis. However, the absence of significant statistical inconsistency does not confirm that the transitivity assumption holds. Transitivity requires that studies contributing to each comparison are sufficiently similar with respect to key effect modifiers, so that indirect comparisons are clinically plausible. Specifically, the included studies varied considerably in several characteristics that may act as effect modifiers and thus threaten transitivity. First, the type of SUD differed across studies: nine studies focused on methamphetamine dependence, one on heroin dependence, and two did not specify the primary substance. Different substances produce distinct neurocognitive impairment patterns, which may modify exercise responses. Second, participant demographics (age, sex, duration of addiction) varied across studies, and these factors are known to influence executive function trajectories and exercise responsiveness. Third, intervention parameters—including exercise intensity, weekly training volume, session duration, and total intervention period—were heterogeneous across studies. Furthermore, owing to the sparsity of the network, most comparisons relied solely on limited direct evidence or entirely on indirect evidence, and most confidence intervals were wide. This may reflect clinical heterogeneity and insufficient direct comparative evidence rather than true statistical consistency; therefore, the relevant results should be interpreted as exploratory estimates only. Overall, the transitivity of the present network is considered clinically fragile. Nevertheless, these findings provide preliminary exploratory evidence for indirect comparisons among different exercise modalities, and their stability warrants further validation through large-scale, multicenter RCTs.

#### 3.5.2 Inhibitory control

The evidence network is shown in Figure 6a, including 12 studies. As shown in Figure 7, compared with TAU, RE [SMD = −1.60, 95%CI (−2.30, −0.91)], MBE [SMD = −1.06, 95%CI (−1.51, −0.61)], and AE [SMD = −0.71, 95%CI (−0.96, −0.46)] significantly improved inhibitory control in Chinese individuals with SUD. Compared to AE, the improvement effect of RE may be more significant [SMD = −0.89, 95%CI (−1.58, −0.21)]. No significant statistical differences were found among the remaining interventions, but all showed certain effect trends. Ranking of interventions in terms of superiority (Figure 8): RE (SUCRA = 96.7%) > MBE (SUCRA = 68.0%) > AE (SUCRA = 35.4%) > TAU (SUCRA = 0.4%).

**Figure 6:**
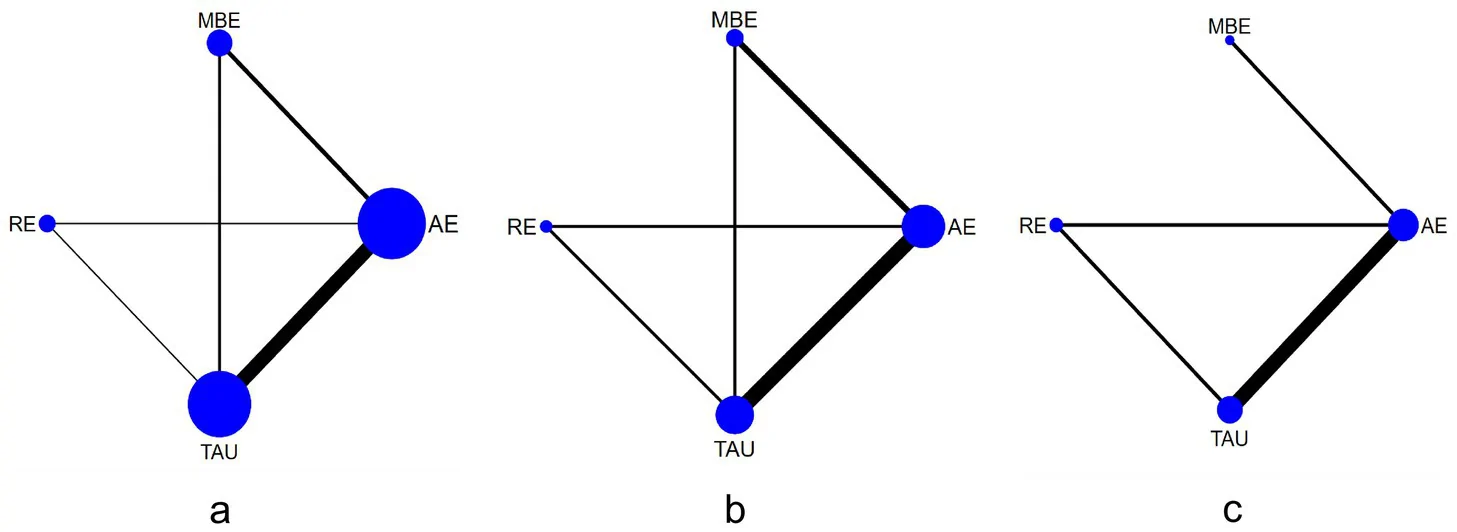
Network evidence map.

**Figure 7:**
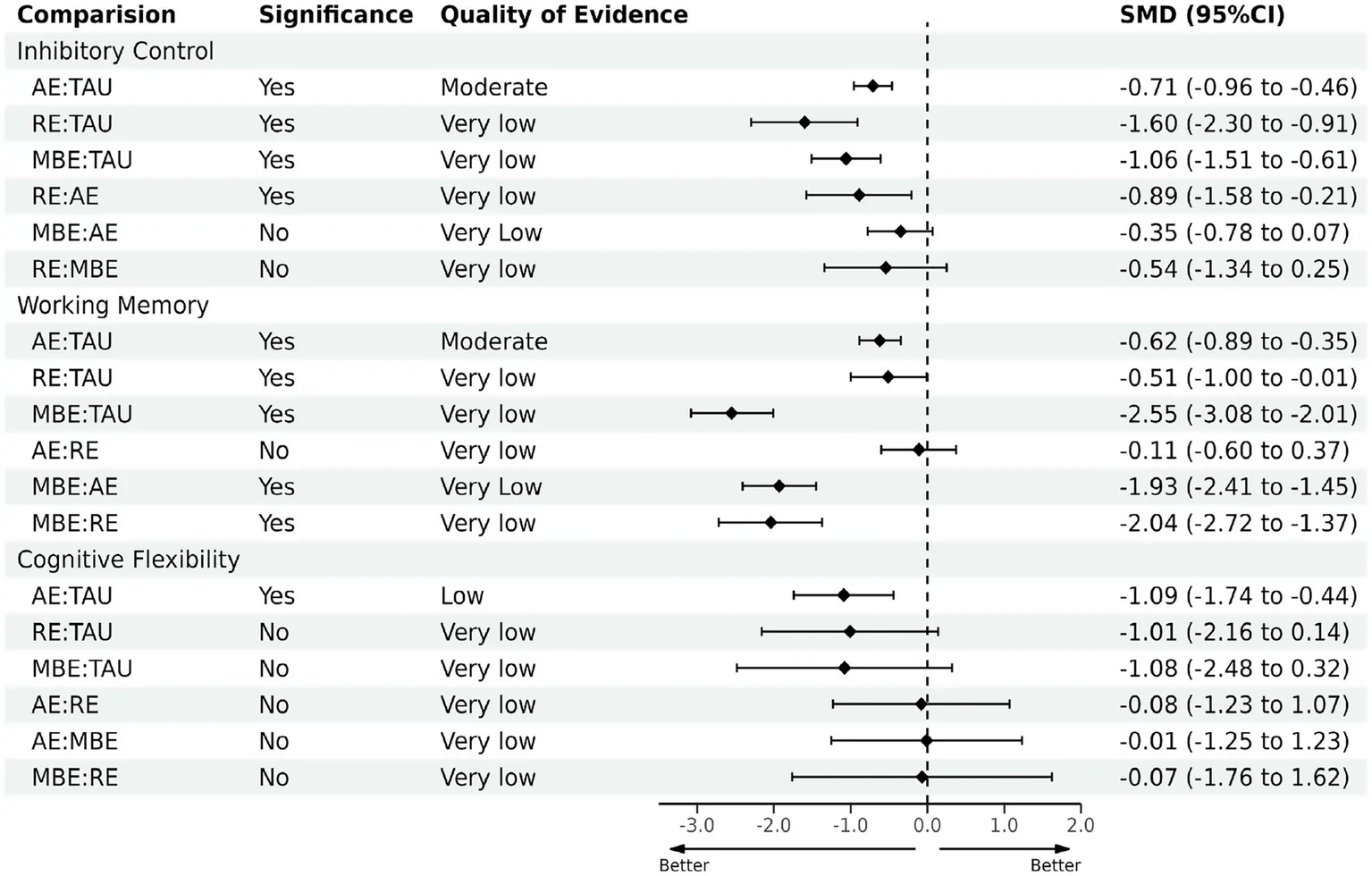
Forest plot of pairwise comparisons. The “Significance” column indicates statistical significance based on whether the 95% confidence intervals cross the line of no effect; the “Quality of Evidence” column provides certainty ratings based on the CINeMA framework.

**Figure 8:**
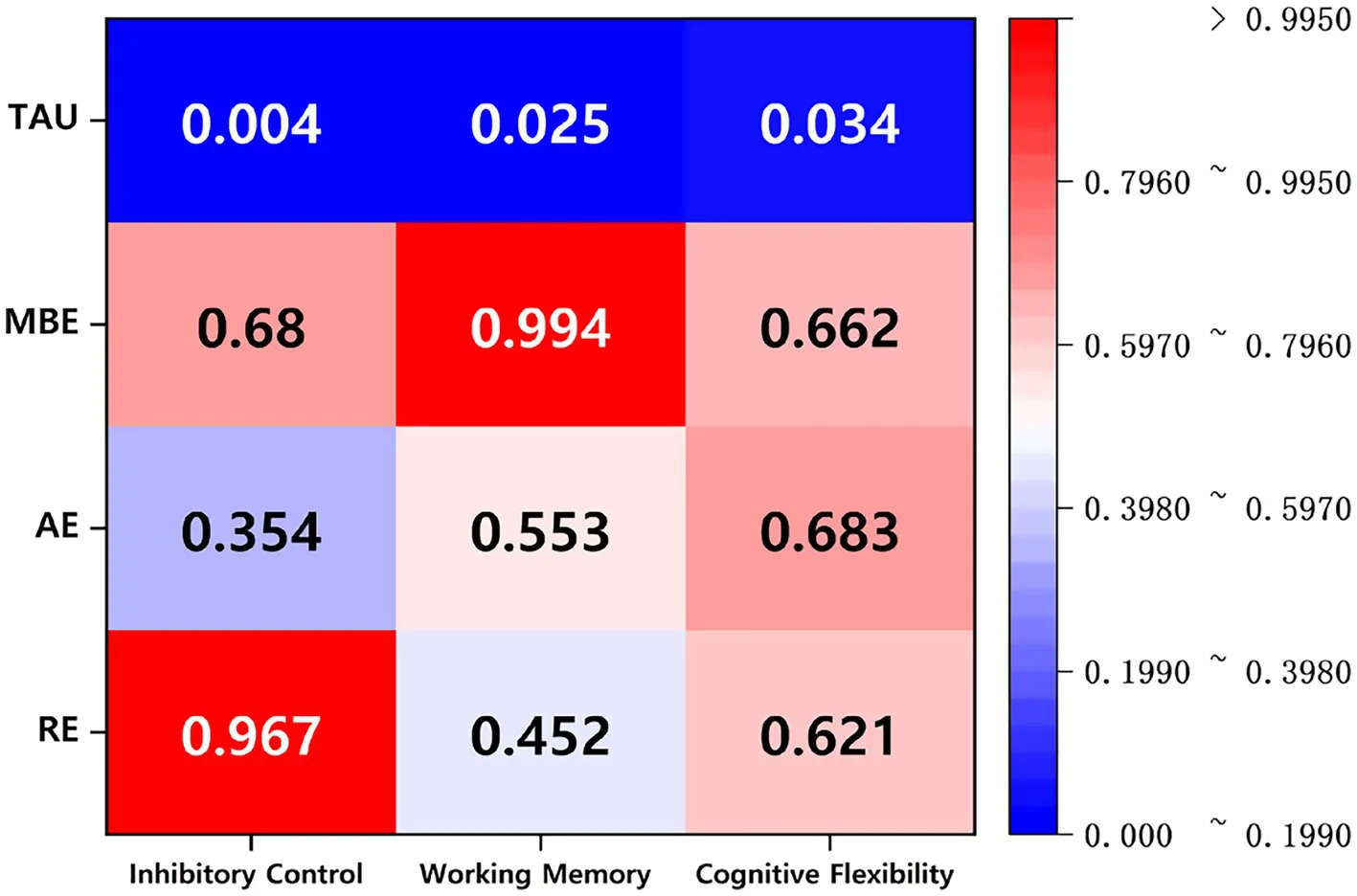
Heatmap of SUCRA values for different exercise modalities across executive function subdomains. The x-axis represents the executive function subdomains, and the y-axis represents the four intervention nodes. The color gradient represents SUCRA values (scaling from 0 to 1).

#### 3.5.3 Working memory

The evidence network is shown in Figure 6b, comprising 6 RCTs. The results (Figure 7) showed that, in terms of working memory, the direct evidence network indicated that MBE [SMD = −2.55, 95% CI (−3.08, −2.01)], AE [SMD = −0.62, 95% CI (−0.89, −0.35)] and RE [SMD = −0.51, 95% CI (−1.00, −0.01)] significantly outperformed TAU, and that MBE was significantly more effective than AE [SMD = −1.93, 95% CI (−2.41, −1.45)]. For the indirect evidence network, MBE was estimated to be significantly superior to RE [SMD = −2.04, 95% CI (−2.72, −1.37)]. No significant statistical differences were found between other interventions. According to the SUCRA ranking probabilities (Figure 8), the order of superiority of the interventions was as follows: MBE (SUCRA = 99.4%) > AE (SUCRA = 55.3%) > RE (SUCRA = 45.2%) > TAU (SUCRA = 2.5%).

#### 3.5.4 Cognitive flexibility

The evidence network is shown in Figure 6c, including 5 RCTs. The results show (Figure 7): In terms of improving cognitive flexibility in Chinese individuals with SUD, AE [SMD = −1.09, 95%CI (−1.74, −0.44)] significantly outperformed TAU, while no statistically significant differences were observed among the other exercise comparisons. The SUCRA ranking is as follows (Figure 8): AE (SUCRA = 68.3%) > MBE (SUCRA = 66.2%) > RE (SUCRA = 62.1%) > TAU (SUCRA = 3.4%).

### 3.6 Quality of evidence assessment

This study employed a conservative CINeMA framework to systematically assess the certainty of evidence for the network meta-analysis across the three executive outcome domains (Table 3). Overall, the certainty of evidence exhibited a highly skewed distribution: only a very few comparisons were rated as “Moderate,” whereas the certainty of all remaining comparisons was “Very low” or “Low.” The primary reasons for downgrading were as follows: First, due to the inherent non-blinding nature of physical exercise interventions, all comparisons were rated as having “Some concerns” or “Major concerns” regarding within-study bias. Second, due to the sparse network structure, the wide confidence intervals of certain comparisons, and the clinically fragile transitivity arising from a high reliance on indirect comparisons, these constituted the main reasons for downgrading due to imprecision. Third, differences in the types of addictive substances, exercise intervention dosages, and measurement tools among individuals with SUD may have caused some concerns regarding heterogeneity; however, most downgrading was not driven by this domain. Fourth, the “Incoherence” domain was rated as “No concerns” for most comparisons; this does not imply that the network has been confirmed to be perfectly consistent, but rather reflects the paucity of direct comparisons, the absence of closed loops, and the low statistical power, which prevented the assessment tool from detecting incoherence. We did not perform additional downgrading based on this domain, but maintained extreme caution when interpreting the network results. Finally, reporting bias was rated as “low risk” overall, indicating that no obvious selective reporting issues were identified.

**Table 3:** Quality of evidence assessment.

Comparison	Studies	Within-study bias	Reporting bias	Indirectness	Imprecision	Heterogeneity	Incoherence	Confidence rating
Inhibitory control								
TAU: AE	10	Some concerns	Low risk	No concerns	No concerns	No concerns	No concerns	Moderate
TAU: RE	1	Major concerns	Low risk	No concerns	Some concerns	No concerns	No concerns	Very low
TAU: MBE	2	Major concerns	Low risk	No concerns	No concerns	No concerns	No concerns	Very low
AE: RE	1	Major concerns	Low risk	No concerns	Some concerns	Some concerns	No concerns	Very low
AE: MBE	3	Major concerns	Low risk	No concerns	No concerns	Some concerns	No concerns	Very Low
RE: MBE	0	Major concerns	Low risk	No concerns	Major concerns	No concerns	Major concerns	Very low
Working memory								
TAU: AE	5	Some concerns	Low risk	No concerns	No concerns	No concerns	No concerns	Moderate
TAU: RE	1	Major concerns	Low risk	No concerns	No concerns	Some concerns	No concerns	Very low
AE: MBE	1	Major concerns	Low risk	No concerns	Some concerns	No concerns	No concerns	Very Low
AE: RE	1	Major concerns	Low risk	No concerns	Major concerns	No concerns	No concerns	Very Low
TAU: MBE	1	Major concerns	Low risk	No concerns	Some concerns	No concerns	No concerns	Very Low
RE: MBE	0	Major concerns	Low risk	No concerns	No concerns	No concerns	Major concerns	Very low
Cognitive flexibility								
TAU: AE	4	Some concerns	Low risk	No concerns	No concerns	Some concerns	No concerns	Low
TAU: RE	1	Major concerns	Low risk	No concerns	Some concerns	Some concerns	No concerns	Very low
AE: MBE	1	Major concerns	Low risk	No concerns	Major concerns	No concerns	Major concerns	Very low
AE: RE	1	Major concerns	Low risk	No concerns	Major concerns	No concerns	No concerns	Very low
TAU: MBE	0	Major concerns	Low risk	No concerns	Major concerns	No concerns	Major concerns	Very low
RE: MBE	0	Major concerns	Low risk	No concerns	Major concerns	No concerns	Major concerns	Very low

In summary, the current evidence suggests that the findings of this study should be regarded as exploratory and hypothesis-generating evidence, which is insufficient to support definitive treatment rankings or clinical recommendations.

### 3.7 Publication bias

The results showed (Figure 9) that the funnel plots for inhibitory control and working memory were relatively symmetrical, indicating no significant publication bias. The funnel plot for cognitive flexibility was asymmetrical, suggesting potential publication bias. Studies for all three indicators fell at the bottom of the funnel plot, indicating a possible small-study effect.

**Figure 9:**
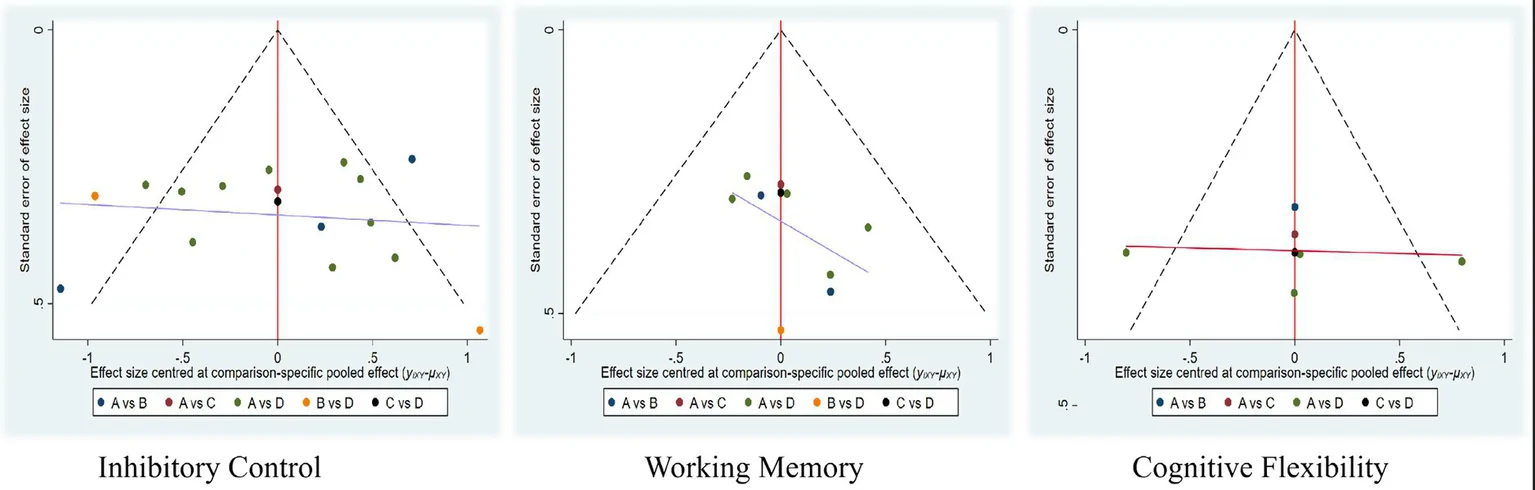
Cognitive flexibility comparison-adjusted funnel plot. A represents AE, B represents MBE, C represents RE, and D represents TAU.

## 4 Discussion

### 4.1 Principal findings

This study first employed a traditional pairwise meta-analysis to directly assess the effects of AE on inhibitory control in Chinese individuals with SUD. The pooled results indicated that, compared with TAU, AE possibly demonstrated a significant advantage in improving inhibitory control, with low statistical heterogeneity across studies. Subgroup analyses based on age, exercise-related factors, and test tools were conducted. The results showed that a single 30-min AE session did not yield a statistically significant effect, whereas the other subgroups demonstrated significant advantages over TAU, with no significant differences between subgroups (I^2^ = 0%).

Subsequently, this study employed, for the first time, a network meta-analysis to systematically compare the chronic intervention effects of different exercise modalities (AE, RE, and MBE) on executive function subdomains in Chinese individuals with SUD. The results revealed exploratory patterns across different exercise types: RE showed a relatively favorable trend for inhibitory control (SUCRA = 96.7%), MBE for working memory (SUCRA = 99.4%), and AE for cognitive flexibility (SUCRA = 68.3%). Pairwise comparisons found that RE may have been significantly superior to AE in improving inhibitory control, and MBE may have been significantly superior to RE and AE in improving working memory. These differential effects may be consistent with the theoretical hypothesis that different exercise modalities exert their roles through distinct neurobiological pathways.

However, it must be emphasized that, although the overall direction of effects is favorable, these findings do not prove the absolute superiority of any specific exercise modality. The significant differences in SUCRA-based rankings and pairwise comparisons stem from a sparse network structure, with most results derived from evidence of “very low” or “low” certainty. Therefore, these findings cannot be used to establish definitive intervention-specific recommendations; rather, they should be regarded solely as exploratory and hypothesis-generating comparative evidence, rather than as a definitive basis for selecting a single optimal intervention. The results aim to provide directional references for future exercise prescription development and the design of large-scale, multicenter confirmatory RCTs. Furthermore, given that all included studies were conducted in China and predominantly involved individuals with methamphetamine use disorder, these findings should be interpreted as applicable primarily to Chinese populations with MA use disorder, and their generalizability to other substance types or geographical contexts remains uncertain.

### 4.2 The chronic effects of different exercise modalities on inhibitory control in individuals with SUD

Meta-analysis results indicated that AE significantly improved inhibitory control in Chinese individuals with SUD compared to TAU. Previous studies have shown that exercise exerts positive effects on addiction recovery, and have suggested that exercise may compensate for dysfunctions in brain reward circuits caused by chronic drug use, thereby mitigating the harmful consequences of substance dependence (Cai and Zhang, 2019). This is consistent with the findings of the present study. The physiological mechanisms may primarily involve normalizing dopaminergic pathways through enhanced cerebral metabolic activity and dopaminergic compensation, thereby regulating redox reactions and alleviating oxidative stress damage, as well as modulating Glu/GABA levels to exert neurobiological effects, ultimately enhancing inhibitory control in individuals with SUD (Lu et al., 2019; Robertson et al., 2016). Furthermore, studies have found that AE not only enhances functional connectivity between the prefrontal and midfrontal regions during the resting state, but also promotes the remodeling and formation of more complex brain networks, thereby enhancing inhibitory control (Chai et al., 2025). These mechanistic studies collectively provide a molecular and neural basis for the restoration of inhibitory control networks.

Subgroup analyses cannot serve as standalone evidence for biological processes, but they can be used to reveal trends and generate hypotheses. In the present study, compared with TAU, AE sessions of 35–40 min and 60 min produced significantly greater intervention effects, whereas the 30-min session did not yield a significant effect. As a form of physical activity, AE primarily regulates neuronal activity through the central nervous system to govern bodily movement; this mechanism also involves the transmission of neurotransmitters, including serotonin, norepinephrine, and dopamine. Evidence suggests that regular or continuous AE lasting 60 min may be the most effective “dose” for promoting remodeling of the mesolimbic dopamine pathway (Weinstock et al., 2017). Furthermore, research on addictive behaviors indicates that longer-duration AE can enhance the brain’s reward system, with exercise duration positively correlated with dopamine release, thereby improving patients’ self-control and ultimately reducing craving and relapse rates (Perreault et al., 2023). A meta-analysis by Chen also indicated that exercise lasting more than 40 min is beneficial for improving executive function in individuals with SUD (Chen et al., 2025). Additionally, some studies recommend rehabilitation training of 35–40 min per session, at least three times per week, for individuals with SUD (Li and Song, 2020), which is consistent with our findings. This recommendation may stem from considerations of practical feasibility and differences in participants’ adaptability; For some individuals with SUD, initiating shorter-duration exercise may be more appropriate, as it can reduce the psychological burden of exercise and increase engagement. It is worth interpreting cautiously that within the 30-min subgroup, after excluding the high-intensity arm of Chen (2021) (the remaining studies were all of moderate intensity), heterogeneity decreased from 50 to 0%. This suggests that the high heterogeneity may be attributable to differences in exercise intensity. Sensitivity analyses showed that excluding any single study did not fundamentally change the direction of the results. However, the 35–40-min subgroup, although demonstrating a larger effect size, was supported by only one study, rendering the result unstable. In summary, any apparent “optimal” dose identified in the subgroup analyses should not be interpreted as an evidence-based recommendation, but rather as a hypothesis-generating observation that requires validation in large-scale trials. Future research should incorporate more high-quality studies to further refine the investigation of specific dose–response relationships, thereby facilitating the development of more individualized rehabilitation programs to improve substance abstinence rates.

The network analysis in this study further revealed that RE exhibited a relatively favorable trend in improving inhibitory control in Chinese individuals with SUD, and its effect may be superior to that of AE. This advantage may be attributed to the unique psychological experiences associated with resistance exercise. Recent evidence suggests that the inherent immediate strength feedback and visible load progression of RE can significantly enhance self-efficacy and psychological resilience, and this positive psychological activation is considered a key mediating factor in improving inhibitory control in individuals with SUD (Nahum et al., 2023). Additionally, studies have indicated that RE may possess unique advantages over AE in improving inhibitory control (Li et al., 2020). From a physiological perspective, the potential advantage of RE may stem from its predominantly anaerobic metabolism characteristics, which might more directly stimulate the release of neurotrophic factors, such as brain-derived neurotrophic factor (BDNF) and insulin-like growth factor-1, thereby enhancing neuroplasticity (Arazi et al., 2021). Concurrently, the complex movement patterns unique to RE—characterized by repetition, high intensity, and multi-joint involvement—may broadly activate neural pathways associated with inhibitory control (Škarabot et al., 2021). However, cautious interpretation is warranted: the overall network structure was relatively sparse, and the significant result for AE versus RE was derived from only one direct comparison with very low certainty of evidence. Therefore, the above findings should be strictly interpreted as “exploratory” and “hypothesis-generating.” Future research requires well-designed, adequately powered direct comparative studies to further validate the robustness of these findings.

### 4.3 The chronic effects of different exercise modalities on working memory in individuals with SUD

The network meta-analysis conducted in this study revealed that MBE exhibited a relatively favorable trend in improving working memory among Chinese individuals with SUD, and its effect may be statistically superior to that of AE and RE. It should be emphasized that this exploratory result does not imply that other forms of exercise are ineffective; rather, the findings also confirmed that both AE and RE were significantly superior to TAU in improving working memory. This suggests that any form of chronic exercise, as a non-pharmacological intervention, may confer overall benefits superior to no exercise, thereby providing robust evidence to support exercise as an adjunctive measure in SUD rehabilitation. Wu et al. demonstrated that MBE can significantly upregulate the expression of brain-derived neurotrophic factor (BDNF), particularly in the hippocampus and prefrontal cortex, promoting neuronal dendritic branching and synaptogenesis while concurrently improving cerebral blood supply and neural connectivity, thereby enhancing memory storage and retrieval (Wu et al., 2013). Most MBE interventions predominantly involve Tai Chi and Qigong. Essentially, these practices are not merely the observation and learning of movements, but rather a form of “dynamic meditation. Previous studies have indicated that Tai Chi and Qigong, as low-load MBE interventions, exert multi-level regulatory effects on the working memory network (Zhang et al., 2026). Furthermore, the beneficial effects of Tai Chi on tasks such as delayed recognition and information updating primarily manifest as enhanced efficiency in the working memory updating process, which depends on strengthened information updating and regulatory functions within relevant neural circuits (Northey et al., 2018). MBE integrates multi-point memory scenes and incorporates characteristics of multiple exercise modalities; the effects of these diverse movement patterns may complement one another, and these features are highly compatible with the demands of working memory tasks. Additionally, a study employing functional near-infrared spectroscopy (fNIRS) found that Tai Chi intervention significantly enhanced right dorsolateral prefrontal cortex (DLPFC) activation and DLPFC–ventrolateral prefrontal cortex (VLPFC) functional connectivity in individuals with SUD through an “intention–qi–form coordination” mechanism. This brain region plays a critical role in the processing, maintenance, and updating of working memory (Wang et al., 2023). The effect of MBE on improving working memory was significantly superior to that observed in the AE group.

However, from the perspective of clinical prescription, the results of this study should not be interpreted as indicating an absolute advantage of MBE in improving working memory among Chinese individuals with SUD. Rather, our findings support a “distribution on demand” rehabilitation concept: for Chinese individuals with SUD characterized by predominant working memory deficits, MBE may be the preferred option. Nevertheless, we must interpret these conclusions with extreme caution. The current network structure lacks direct comparative studies among different exercise modalities; the superiority exhibited by MBE relies to some extent on indirect comparisons—albeit with a larger statistically significant effect size—and some results are heavily dependent on indirect evidence transmitted through TAU. In such a sparse network, statistical power is low, and the certainty of evidence is mostly rated as “very low” or “low.” Therefore, the above conclusions should be regarded as exploratory and hypothesis-generating, and their specific mechanisms and clinical applicability warrant focused attention and in-depth investigation through future large-scale, multicenter, head-to-head RCTs.

### 4.4 The chronic effects of different exercise modalities on cognitive flexibility in individuals with SUD

For cognitive flexibility, the network meta-analysis indicated a relative advantage of AE over other modalities, and its effect may be statistically superior to TAU. However, it is important to note that, compared with TAU, RE and MBE did not yield statistically significant improvements. Cognitive flexibility requires multidimensional synergy across neural structures, neurotransmitter regulation, and dynamic information processing to efficiently accomplish information switching, task shifting, and environmental adaptation, with adequate cerebral blood flow serving as the foundation for its optimal functioning. Individuals with SUD often exhibit blunted prefrontal hemodynamic responses during exercise, suggesting that their cognitive flexibility may rely more heavily on exercise-induced restoration of prefrontal perfusion and improvements in network efficiency. The American Heart Association (AHA) has noted that regular AE increases the diameter of major cerebral arteries, thereby enhancing blood flow and providing more sufficient oxygen and nutrients to brain regions closely related to cognitive flexibility, such as the prefrontal cortex (PFC), ensuring normal brain metabolic activity (Kleinloog et al., 2019). A meta-analysis by Ludyga et al. (2016) demonstrated that participants exhibited significant improvements in cognitive flexibility tasks, with reduced error rates and faster reaction times after AE. Furthermore, a large body of epidemiological evidence confirms that non-SUD populations adhering to long-term AE have a significantly reduced risk of cognitive decline and better maintenance of cognitive flexibility (Mortimer et al., 2012; Zheng et al., 2016). Although study populations varied, the observed intervention effects were relatively consistent, suggesting the potential for cross-population efficacy of AE in improving cognitive flexibility. In contrast, studies on MBE regarding cognitive flexibility are relatively scarce, and most of them focus on physical health and mental health research (Smelson et al., 2013; Lu et al., 2026; Li et al., 2013). Similarly, while RE demonstrates unique advantages in inhibitory control, its anaerobic mechanism centered on strength loading may lack targeted stimulation for dynamic cerebral perfusion and neuroplastic remodeling of relevant brain regions, which are essential for the rapid switching and dynamic adaptation required in cognitive flexibility tasks (Landrigan et al., 2020; Herold et al., 2019). It is worth noting, however, that these mechanistic explanations are primarily derived from theoretical assumptions based on broader populations and should therefore be interpreted with caution. Currently, specific mechanistic explorations into how exercise improves cognitive flexibility in individuals with SUD remain relatively immature, and direct empirical evidence within this population is still limited.

However, extreme caution is still warranted when interpreting the above conclusions. The number of included studies directly comparing RE and MBE on cognitive flexibility was extremely small, resulting in a highly sparse network structure; some comparisons even completely lacked direct evidence and relied solely on indirect evidence via TAU. Therefore, the non-significant results for RE and MBE should not be simply interpreted as “ineffective,” but are more likely attributable to “imprecision” caused by insufficient evidence. In summary, the observed advantage of AE in cognitive flexibility should be regarded as preliminary, exploratory hypothesis-generating evidence; its specific mechanisms and clinical applicability urgently warrant focused attention and in-depth investigation through large-sample, high-quality RCTs in the future.

### 4.5 Limitations

First, the number of RCTs included for some interventions was relatively small, and the statistical power for both direct and indirect comparisons was limited. Consequently, the current evidence base may not be robust enough to support a large-scale meta-analysis for fitting dose–response relationships or deriving a stable exercise prescription. Second, the network structure of the included studies is sparse, and clinical transitivity is fragile, resulting in insufficient precision and stability of the results. Third, all included primary studies reported only immediate post-intervention effects and lacked long-term follow-up data, thus precluding the evaluation of the long-term effects of exercise interventions. Fourth, owing to the limited number of available primary studies, we were unable to subdivide exercise modalities into more specific subtypes, thereby precluding the assessment of potential intra-modality differences in treatment effects. Fifth, this study only included RCT studies published since the database was established and did not obtain or include unpublished literature. Sixth, all included studies were conducted in China, and most participants had methamphetamine or heroin use disorders. Two studies did not clearly report the primary substance of abuse. This limits the generalizability of the findings to other countries, cultural contexts, treatment systems, and substance types. In particular, the effects of different exercise modalities on executive function may vary across substances because of differences in neurocognitive impairment patterns, treatment environments, and patterns of polysubstance use. Therefore, the findings of this analysis may be primarily applicable to individuals with MA use disorder in China, and may not be generalizable to individuals with SUD outside of China or to users of other substances. Seventh, inhibitory control, working memory, and cognitive flexibility are theoretically distinct but closely interlinked executive function domains that are often measured within the same participants and may share common underlying neural substrates. The present analysis treated these domains as independent outcomes, which may not fully account for potential correlations between them or the statistical dependence arising from repeated measurements within the same studies. This approach may increase the risk of Type I error due to multiple comparisons.

## 5 Conclusion

This systematic review and exploratory network meta-analysis integrated traditional pairwise and network evidence and found that different exercise interventions exert beneficial effects on executive function–related outcomes in Chinese individuals with SUD. Specifically, traditional meta-analysis indicated that AE has a positive impact on overall inhibitory control, but subgroup analysis revealed that a single 30-min session possibly produced no significant effect. Network meta-analysis further revealed potential, exploratory advantages of different exercise modalities across distinct executive function subdomains: RE presents a relatively favorable trend in inhibitory control (SUCRA = 96.7%), MBE may present a relatively favorable trend in working memory (SUCRA = 99.4%), and AE may present a relatively favorable trend in cognitive flexibility (SUCRA = 68.3%). Pairwise comparisons suggested that RE might be superior to AE in improving inhibitory control, and MBE might be superior to both AE and RE in improving working memory. These exploratory rankings should not be overinterpreted as definitive efficacy hierarchies.

However, due to limitations in network size and evidence quality, the ability of this study to infer definitive clinically beneficial rankings was constrained to some extent. The current evidence is insufficient to establish reliable rankings of intervention superiority, a single optimal exercise strategy, or the absolute preeminence of specific exercise prescription parameters. Future network meta-analyses should incorporate studies from diverse regions and clearly distinguish substance types to enhance the generalizability of the results; meanwhile, future trials should prioritize large-sample, multicenter RCTs that thoroughly evaluate different dose–response relationships and precisely compare the advantages of various exercise modalities within well-defined SUD subgroups, so as to further validate the exploratory findings of this study.

## Data Availability

The original contributions presented in the study are included in the article/Supplementary material, further inquiries can be directed to the corresponding author.

## References

[ref1] Albinet C. T. Boucard G. Bouquet C. A. Audiffren M. (2012). Processing speed and executive functions in cognitive aging: how to disentangle their mutual relationship? Brain Cogn. 79, 1–11. doi: 10.1016/j.bandc.2012.02.001, 22387275

[ref2] Anzures-Cabrera J. Higgins J. P. (2010). Graphical displays for meta-analysis: an overview with suggestions for practice. Res. Synth. Methods 1, 66–80. doi: 10.1002/jrsm.6, 26056093

[ref3] Arazi H. Babaei P. Moghimi M. Asadi A. (2021). Acute effects of strength and endurance exercise on serum BDNF and IGF-1 levels in older men. BMC Geriatr. 21:50. doi: 10.1186/s12877-020-01937-6, 33441099 PMC7807435

[ref4] Bahji A. (2025). Limitations and future directions in pharmacological treatment for amphetamine-type stimulant use disorder. Can. J. Psychiatr. 70, 136–137. doi: 10.1177/07067437241312683, 39819145 PMC11748372

[ref5] Battle D. E. (2013). Diagnostic and statistical manual of mental disorders (DSM). Codas 25, 190–191. doi: 10.1590/s2317-17822013000200017, 24413388

[ref6] Cai C. X. Zhang Y. L. (2019). Research progress on the mechanism of exercise improving brain executive function. J. Chengdu Sport Univ. 45, 120–126. doi: 10.15942/j.jcsu.2019.06.020

[ref7] Ceceli A. O. King S. G. McClain N. Alia-Klein N. Goldstein R. Z. (2023). The neural signature of impaired inhibitory control in individuals with heroin use disorder. J. Neurosci. 43, 173–182. doi: 10.1523/JNEUROSCI.1237-22.2022, 36396402 PMC9838696

[ref8] Cefis M. Chaney R. Wirtz J. Méloux A. Quirié A. Leger C. . (2023). Molecular mechanisms underlying physical exercise-induced brain BDNF overproduction. Front. Mol. Neurosci. 16:1275924. doi: 10.3389/fnmol.2023.1275924, 37868812 PMC10585026

[ref9] Chai Q. Y. Song A. Q. Zhao Q. Y. Shen Q. Q. Cui L. (2025). An ALE meta-analysis on the effects of neural changes due to exercise on executive function in a healthy population. Sci. Rep. 15:34415. doi: 10.1038/s41598-025-17431-1, 41039062 PMC12491555

[ref10] Chang Y. K. Labban J. D. Gapin J. I. Etnier J. L. (2012). The effects of acute exercise on cognitive performance: a meta-analysis. Brain Res. 1453, 87–101. doi: 10.1016/j.brainres.2012.02.068, 22480735

[ref11] Chen Y. Liu T. Zhou C. (2021). Effects of 12-week aerobic exercise on cue-induced drug craving in methamphetamine-dependent patients and the moderation effect of working memory. Ment. Health Phys. Act. 21:100420. doi: 10.1016/j.mhpa.2021.100420

[ref12] Chen J. Wang X. Zhang X. Xiao W. Kabachkova A. Tan Y. (2025). Effects of exercise on executive function in individuals with drug addiction: a systematic review and three-level meta-analysis. Front. Sports Act. Living 7:1646327.13. doi: 10.3389/fspor.2025.1646327, 41323873 PMC12657471

[ref13] Cumpston M. Li T. Page M. J. Chandler J. Welch V. A. Higgins J. P. T. . (2019). Updated guidance for trusted systematic reviews: a new edition of the Cochrane handbook for systematic reviews of interventions. Cochrane Database Syst. Rev. 10:ED000142. doi: 10.1002/14651858.ED000142, 31643080 PMC10284251

[ref14] Edwards J. J. Deenmamode A. H. P. Griffiths M. Arnold O. Cooper N. J. Wiles J. D. . (2023). Exercise training and resting blood pressure: a large-scale pairwise and network meta-analysis of randomised controlled trials. Br. J. Sports Med. 57, 1317–1326. doi: 10.1136/bjsports-2022-106503, 37491419

[ref15] Fang C. L. Zheng G. H. (2024). Impact of physical exercise on the executive function and mental health of heroin addicts. Exerc. Sci. 33, 477–493. doi: 10.15857/ksep.2024.00402

[ref16] He Y. J. (2022). Effects of a 16-week Basketball Exercise Intervention on Executive Function in Individuals Undergoing Compulsory Drug Rehabilitation. Taiyuan, China: Shanxi Normal University. doi: 10.27287/d.cnki.gsxsu.2022.000997

[ref17] He M. Yang S. Miao Y. Zhang W. Zhu D. Xu D. (2021). Four-week tai chi intervention decreases attention bias to drug cues in individuals with methamphetamine use disorder. Am. J. Drug Alcohol Abuse 47, 638–648. doi: 10.1080/00952990.2021.1950745, 34325579

[ref18] Herold F. Törpel A. Schega L. Müller N. G. (2019). Functional and/or structural brain changes in response to resistance exercises and resistance training lead to cognitive improvements - a systematic review. Eur. Rev. Aging Phys. Act. 16:10. doi: 10.1186/s11556-019-0217-2, 31333805 PMC6617693

[ref19] Kleinloog J. P. D. Mensink R. P. Ivanov D. Adam J. J. Uludağ K. Joris P. J. (2019). Aerobic exercise training improves cerebral blood flow and executive function: a randomized, controlled cross-over trial in sedentary older men. Front. Aging Neurosci. 11:333. doi: 10.3389/fnagi.2019.00333, 31866855 PMC6904365

[ref20] Koob G. F. Volkow N. D. (2010). Neurocircuitry of addiction. Neuropsychopharmacology 35, 217–238. doi: 10.1038/npp.2009.110, 19710631 PMC2805560

[ref21] Landrigan J. F. Bell T. Crowe M. Clay O. J. Mirman D. (2020). Lifting cognition: a meta-analysis of effects of resistance exercise on cognition. Psychol. Res. 84, 1167–1183. doi: 10.1007/s00426-019-01145-x, 30627769

[ref22] Li L. Cui J. Xiang Q. . (2020). The effect of 8-week different types of exercise on executive function in female college students. Chin. J. Sports Med. 39, 810–816. doi: 10.16038/j.1000-6710.2020.10.010

[ref23] Li Y. L. Song E. (2020). Expert consensus on exercise prescription for drug rehabilitation personnel. Chin. J. Sports Med. 39, 837–844. doi: 10.16038/j.1000-6710.2020.11.001

[ref24] Li D. X. Zhuang X. Y. Zhang Y. P. Guo H. Wang Z. Zhang Q. . (2013). Effects of tai chi on the protracted abstinence syndrome: a time trial analysis. Am. J. Chin. Med. 41, 43–57. doi: 10.1142/S0192415X13500043, 23336506

[ref25] Liu X. X. (2021). The Effect of Aerobic Exercise on Executive Function in Methamphetamine Dependents. Fuzhou China: Fujian Normal University. doi: 10.27019/d.cnki.gfjsu.2021.001368

[ref26] Liu X. Wang S. (2023). Effects of aerobic exercise combined with attentional bias training on cognitive function and psychiatric symptoms of individuals with methamphetamine dependency: a randomized controlled trial. Int. J. Ment. Health Addict. 21, 1727–1745. doi: 10.1007/s11469-021-00686-w

[ref27] Lu C. Dong W. Zheng L. Zhang J. Pan J. Liu Y. . (2019). Effects of exercise intervention on activating immune stress and dopamine levels on mental health of compulsory isolated detoxification personnel with amphetamine-type stimulant addiction. Chin. J. Sports Med. 38, 762–770. doi: 10.16038/j.1000-6710.2019.09.005

[ref28] Lu M. Y. Wang X. Chen J. W. . (2026). Effects of traditional health-preserving exercises on psychological health intervention for drug addicts: a meta-analysis. Chin. J. Drug Depend. 35, 152–159. doi: 10.13936/j.cnki.cjdd1992.2026.02.013

[ref29] Ludyga S. Gerber M. Brand S. Holsboer-Trachsler E. Pühse U. (2016). Acute effects of moderate aerobic exercise on specific aspects of executive function in different age and fitness groups: a meta-analysis. Psychophysiology 53, 1611–1626. doi: 10.1111/psyp.12736, 27556572

[ref30] Menglu S. Ruiwen L. Suyong Y. Dong Z. (2021). Effects of tai chi on the executive function and physical fitness of female methamphetamine dependents: a randomized controlled trial. Front. Psych. 12:653229. doi: 10.3389/fpsyt.2021.653229, 34177646 PMC8222617

[ref31] Miyake A. Friedman N. P. Emerson M. J. Witzki A. H. Howerter A. Wager T. D. (2000). The unity and diversity of executive functions and their contributions to complex "frontal lobe" tasks: a latent variable analysis. Cogn. Psychol. 41, 49–100. doi: 10.1006/cogp.1999.0734, 10945922

[ref32] Mortimer J. A. Ding D. Borenstein A. R. DeCarli C. Guo Q. Wu Y. . (2012). Changes in brain volume and cognition in a randomized trial of exercise and social interaction in a community-based sample of non-demented Chinese elders. J. Alzheimer's Dis 30, 757–766. doi: 10.3233/JAD-2012-120079, 22451320 PMC3788823

[ref33] Munn Z. Stern C. Aromataris E. Lockwood C. Jordan Z. (2018). What kind of systematic review should I conduct? A proposed typology and guidance for systematic reviewers in the medical and health sciences. BMC Med. Res. Methodol. 18:5. doi: 10.1186/s12874-017-0468-4, 29316881 PMC5761190

[ref34] Nahum M. Sinvani R. T. Afek A. Ben Avraham R. Jordan J. T. Ben Shachar M. S. . (2023). Inhibitory control and mood in relation to psychological resilience: an ecological momentary assessment study. Sci. Rep. 13:13151. doi: 10.1038/s41598-023-40242-1, 37573400 PMC10423230

[ref35] Northey J. M. Cherbuin N. Pumpa K. L. Smee D. J. Rattray B. (2018). Exercise interventions for cognitive function in adults older than 50: a systematic review with meta-analysis. Br. J. Sports Med. 52, 154–160. doi: 10.1136/bjsports-2016-096587, 28438770

[ref36] Page M. J. McKenzie J. E. Bossuyt P. M. Boutron I. Hoffmann T. C. Mulrow C. D. . (2020). Updating guidance for reporting systematic reviews: development of the PRISMA 2020 statement. J. Clin. Epidemiol. 134, 103–112. doi: 10.1016/j.jclinepi.2021.02.003, 33577987

[ref37] Perreault B. Hammond N. Thanos P. K. (2023). Effects of exercise on testosterone and implications of drug abuse: a review. Clin. Neuropharmacol. 46, 112–122. doi: 10.1097/WNF.0000000000000546, 37191565

[ref38] Potenza M. N. (2014). The neural bases of cognitive processes in gambling disorder. Trends Cogn. Sci. 18, 429–438. doi: 10.1016/j.tics.2014.03.007, 24961632 PMC4112163

[ref39] Robertson C. L. Ishibashi K. Chudzynski J. Mooney L. J. Rawson R. A. Dolezal B. A. . (2016). Effect of exercise training on striatal dopamine D2/D3 receptors in methamphetamine users during behavioral treatment. Neuropsychopharmacology 41, 1629–1636. doi: 10.1038/npp.2015.331, 26503310 PMC4832026

[ref40] Škarabot J. Brownstein C. G. Casolo A. Del Vecchio A. Ansdell P. (2021). The knowns and unknowns of neural adaptations to resistance training. Eur. J. Appl. Physiol. 121, 675–685. doi: 10.1007/s00421-020-04567-3, 33355714 PMC7892509

[ref41] Smelson D. Chen K. W. Ziedonis D. Andes K. Lennox A. Callahan L. . (2013). A pilot study of qigong for reducing cocaine craving early in recovery. J. Altern. Complement. Med. 19, 97–101. doi: 10.1089/acm.2012.0052, 22757968 PMC3576894

[ref42] Sterne J. A. C. Savović J. Page M. J. Elbers R. G. Blencowe N. S. Boutron I. . (2019). RoB 2: a revised tool for assessing risk of bias in randomised trials. BMJ 366:l4898. doi: 10.1136/bmj.l4898, 31462531

[ref43] UNODC (2025). World Drug Report 2025. New York: United Nations publication.

[ref44] Volkow N. D. Fowler J. S. Wang G. J. Swanson J. M. (2004). Dopamine in drug abuse and addiction: results from imaging studies and treatment implications. Mol. Psychiatry 9, 557–569. doi: 10.1038/sj.mp.4001507, 15098002

[ref45] Volkow N. D. Koob G. F. McLellan A. T. (2016). Neurobiologic advances from the brain disease model of addiction. N. Engl. J. Med. 374, 363–371. doi: 10.1056/NEJMra1511480, 26816013 PMC6135257

[ref46] Wang C. Dai Y. Yang Y. Yuan X. Zhang M. Zeng J. . (2023). Effects of tai chi on working memory in older adults: evidence from combined fNIRS and ERP. Front. Aging Neurosci. 15:1206891. doi: 10.3389/fnagi.2023.1206891, 37455937 PMC10340122

[ref47] Wang D. Wang Y. Wang Y. Li R. Zhou C. (2014). Impact of physical exercise on substance use disorders: a meta-analysis. PLoS One 9:e110728. doi: 10.1371/journal.pone.0110728, 25330437 PMC4199732

[ref48] Wang D. Zhu T. Zhou C. Chang Y.-K. (2017). Aerobic exercise training ameliorates craving and inhibitory control in methamphetamine dependencies: a randomized controlled trial and event-related potential study. Psychol. Sport Exerc. 30, 82–90. doi: 10.1016/j.psychsport.2017.02.001

[ref49] Weinstock J. Farney M. R. Elrod N. M. Henderson C. E. Weiss E. P. (2017). Exercise as an adjunctive treatment for substance use disorders: rationale and intervention description. J. Subst. Abus. Treat. 72, 40–47. doi: 10.1016/j.jsat.2016.09.002, 27666958 PMC5289308

[ref50] World Health Organization (2019). Global action plan on Physical Activity 2018–2030: more Active People for a Healthier World. Geneva: World Health Organization.

[ref51] Wu Y. Wang Y. Burgess E. O. Wu J. (2013). The effects of tai chi exercise on cognitive function in older adults: a meta-analysis. J. Sport Health Sci. 2, 193–203. doi: 10.1016/j.jshs.2013.09.001

[ref52] Xu X. Y. (2025). Study on the Intervention Effect of Tai Chi on Cognitive Function of Drug Rehabilitation Personnel: Evidence Based on Near-Infrared Spectroscopy Imaging. Shanghai University of Sport. doi: 10.27315/d.cnki.gstyx.2025.000271

[ref53] Yates J. R. (2024). Pharmacological treatments for methamphetamine use disorder: current status and future targets. Subst. Abus. Rehabil. 15, 125–161. doi: 10.2147/SAR.S431273, 39228432 PMC11370775

[ref54] Zhang L. Li Y. Wang L. Zhang X. Sun Y. He S. . (2026). Effects of tai chi on attentional bias in men with methamphetamine use disorder. NPJ Sci. Learn. 11:40. doi: 10.1038/s41539-026-00426-6, 42000754 PMC13276401

[ref55] Zhang Y. H. Tang A. Zhou C. L. . (2024). Effects of rehabilitation exercise intervention on inhibitory control in female methamphetamine addicts: an event-related potential study. Chin. J. Rehabil. Med. 39, 201–209. doi: 10.3969/j.issn.1001-1242.2024.02.009

[ref56] Zheng G. Xia R. Zhou W. Tao J. Chen L. (2016). Aerobic exercise ameliorates cognitive function in older adults with mild cognitive impairment: a systematic review and meta-analysis of randomised controlled trials. Br. J. Sports Med. 50, 1443–1450. doi: 10.1136/bjsports-2015-095699, 27095745

[ref57] Zhu T. Tao W. Peng B. Su R. Wang D. Hu C. . (2022). Effects of a group-based aerobic exercise program on the cognitive functions and emotions of substance use disorders patients: a randomized controlled trial. Int. J. Ment. Health Addict. 20, 2349–2365. doi: 10.1007/s11469-021-00518-x

[ref58] Zhu Y. Zhu J. Song G. (2023). The impact of aerobic exercise training on cognitive function and gut microbiota in methamphetamine-dependent individuals in the community. Physiol. Behav. 270:114302. doi: 10.1016/j.physbeh.2023.114302, 37474085

