# Supplementary material for: Chronic effects of different exercise modalities on executive function in Chinese individuals with substance use disorders: a systematic review and network meta-analysis
Source: Front Psychol. 2026 Oct 27;17:1979254. doi: 10.3389/fpsyg.2026.1979254 (PMC13761228; doi:10.3389/fpsyg.2026.1979254)
Supplement: Supplementary file 2 [file Supplementary_file_2.DOCX]

**WOC and MEDLINE**

**#1 TS=(Exercise OR "Physical Exercise" OR "Isometric Exercise" OR "Aerobic Exercise" OR "Exercise Training" OR "Physical Activity" OR "Physical Activities" OR "Active Breaks" OR "Activity Breaks" OR "Acute Exercise" OR "Resistance Training" OR "Resistance train" OR "Resistance exercise" OR "High Intensity Interval Training" OR HIIT OR "High-Intensity Interval Training" OR "Mind body exercise" OR "Chinese exercise" OR "traditional exercise" OR "Chinese traditional exercise" OR "Traditional Chinese Exercise" OR "exercise" OR "activity")**

**#2 TS=("Substance-Related Disorders" OR "Drug Habituation" OR "Drug Use Disorder" OR "Substance Abuse" OR "Substance Dependence" OR "Chemical Dependence" OR "Substance Addiction" OR "Substance Use Disorder" OR "Drug Dependence" OR "Drug Addiction" OR "Drug Abuse, Prescription" OR "Drug Abuse" OR Amphetamine OR Cocaine OR Heroin OR Methadone OR Morphine OR Opioid OR Cannabis)**

**#3 TS=("executive function" OR "Executive Control" OR "inhibitory control" OR "working memory" OR "cognitive flexibility")**

**#1 AND #2 AND #3**

**Embase**

**#1 'exercise'/exp OR 'exercise' OR 'aerobic exercise'/exp OR 'aerobic exercise' OR 'resistance training'/exp OR 'resistance training' OR 'high intensity interval training'/exp OR 'high intensity interval training' OR 'mind body exercise'/exp OR 'mind body exercise' OR 'physical activity'/exp OR 'physical activity' OR exercise:ti,ab,kw OR 'physical exercise':ti,ab,kw OR 'isometric exercise':ti,ab,kw OR 'aerobic exercise':ti,ab,kw OR 'exercise training':ti,ab,kw OR 'physical activity':ti,ab,kw OR 'physical activities':ti,ab,kw OR 'active breaks':ti,ab,kw OR 'activity breaks':ti,ab,kw OR 'acute exercise':ti,ab,kw OR 'resistance training':ti,ab,kw OR 'resistance train':ti,ab,kw OR 'resistance exercise':ti,ab,kw OR 'high intensity interval training':ti,ab,kw OR hiit:ti,ab,kw OR 'high-intensity interval training':ti,ab,kw OR 'mind body exercise':ti,ab,kw OR 'chinese exercise':ti,ab,kw OR 'traditional exercise':ti,ab,kw OR 'chinese traditional exercise':ti,ab,kw OR 'traditional chinese exercise':ti,ab,kw**

**#2 'substance abuse'/exp OR 'drug dependence'/exp OR 'drug addiction'/exp OR 'substance use disorder'/exp OR 'amphetamine'/exp OR 'cocaine'/exp OR 'heroin'/exp OR 'methadone'/exp OR 'morphine'/exp OR 'opioid'/exp OR 'cannabis'/exp OR 'substance-related disorders':ti,ab,kw OR 'drug habituation':ti,ab,kw OR 'drug use disorder':ti,ab,kw OR 'substance abuse':ti,ab,kw OR 'substance dependence':ti,ab,kw OR 'chemical dependence':ti,ab,kw OR 'substance addiction':ti,ab,kw OR 'substance use disorder':ti,ab,kw OR 'drug dependence':ti,ab,kw OR 'drug addiction':ti,ab,kw OR 'drug abuse, prescription':ti,ab,kw OR 'drug abuse':ti,ab,kw OR amphetamine:ti,ab,kw OR cocaine:ti,ab,kw OR heroin:ti,ab,kw OR methadone:ti,ab,kw OR morphine:ti,ab,kw OR opioid:ti,ab,kw OR cannabis:ti,ab,kw**

**#3 'executive function'/exp OR 'inhibitory control'/exp OR 'working memory'/exp OR 'cognitive flexibility'/exp OR 'executive function':ti,ab,kw OR 'executive control':ti,ab,kw OR 'inhibitory control':ti,ab,kw OR 'working memory':ti,ab,kw OR 'cognitive flexibility':ti,ab,kw**

**#1 AND #2 AND #3**

**Cochrane library**

**ID	Search	Hits**

**#1	MeSH descriptor: [Exercise] explode all trees	43817**

**#2	(Exercise, Physical):ti,ab,kw OR (Physical Exercise):ti,ab,kw OR (Exercise, Isometric):ti,ab,kw**

**OR (Isometric Exercise):ti,ab,kw OR (Aerobic Exercise):ti,ab,kw	79890**

**#3	(Exercise, Aerobic):ti,ab,kw OR (activity):ti,ab,kw OR (Training, Exercise):ti,ab,kw OR**

**(Physical Activity):ti,ab,kw OR (Activities, Physical):ti,ab,kw	271996**

**#5	(Acute Exercise):ti,ab,kw OR (Resistance train):ti,ab,kw OR (Resistance exercise):ti,ab,kw OR**

**(High Intensity Interval Training):ti,ab,kw OR (HIIT):ti,ab,kw	38242**

**#6	(Mind body exercise):ti,ab,kw OR (Chinese exercise):ti,ab,kw OR (traditional exercise):ti,ab,kw**

**OR (High-Intensity Interval Training):ti,ab,kw OR (Traditional Chinese Exercise):ti,ab,kw	13511**

**#7	#1 OR #2 OR #3 OR #5 OR #6	308384**

**#8	MeSH descriptor: [Substance-Related Disorders] explode all trees	22400**

**#9	(Disorder, Substance Related):ti,ab,kw OR (Related Disorder, Substance):ti,ab,kw**

**OR (Drug Habituation):ti,ab,kw OR (Drug Use Disorder):ti,ab,kw**

**OR (Substance Abuse):ti,ab,kw	36376**

**#10	(Substance Dependence):ti,ab,kw OR (Chemical Dependence):ti,ab,kw**

**OR (Substance Addiction):ti,ab,kw OR (Substance Use Disorder):ti,ab,kw**

**OR (Drug Dependence):ti,ab,kw	14981**

**#11	(Drug Addiction):ti,ab,kw OR (Drug Abuse):ti,ab,kw OR (Amphetamine):ti,ab,kw**

**OR (Cocaine):ti,ab,kw OR (Heroin):ti,ab,kw	18404**

**#12	(Methadone):ti,ab,kw OR (morphine):ti,ab,kw OR (Opioid):ti,ab,kw OR (Cannabis):ti,ab,kw	51024**

**#13	#8 OR #9 OR #10 OR #11 OR #12	104160**

**#14	MeSH descriptor: [Executive Function] explode all trees	2159**

**#15	(Function, Executive):ti,ab,kw OR (Executive Control):ti,ab,kw OR (Inhibitory control):ti,ab,kw**

**OR (Working memory):ti,ab,kw OR (Cognitive flexibility):ti,ab,kw	23408**

**#16	#14 OR #15	23408**

**#17	#7 AND #13 AND #16	422**

**Inhibitory control**

#1 network setup mean sd n, study(id) trt(t) format(augment) smd

#2 network map, improve

#3 network meta i

#4 network meta c

#5 network forest

#6 network sidesplit all, tau

#7 clear

#8 network setup mean sd n, study(id) trt(t) format(augment) smd

#9 network meta i

#10 network meta c

#11 network rank min, all zero reps(5000) gen(prob)

#12 sucra prob*, lab(AE RE MBE TAU)

#13 netleague, lab(AE RE MBE TAU) sort(RE MBE AE TAU)

#14 intervalplot, null(0) lab(AE RE MBE TAU)

#15 network convert pairs

#16 netfunnel _y _stderr _t1 _t2 , random bycomp add(lfit _stderr _ES_CEN) noalpha

#17 ifplot _y _stderr _t1 _t2 id, tau2(loop)

**Working memory**

#1 network setup mean sd n, study(id) trt(t) format(augment) smd

#2 network map, improve

#3 network meta i

#4 network meta c

#5 network forest

#6 network sidesplit all, tau

#7 clear

#8 network setup mean sd n, study(id) trt(t) format(augment) smd

#9 network meta i

#10 network meta c

#11 network rank min, all zero reps(5000) gen(prob)

#12 sucra prob*, lab(AE RE MBE TAU)

#13 netleague, lab(AE RE MBE TAU) sort(MBE AE RE TAU)

#14 intervalplot, null(0) lab(AE RE MBE TAU)

#15 network convert pairs

#16 netfunnel _y _stderr _t1 _t2 , random bycomp add(lfit _stderr _ES_CEN) noalpha

#17 ifplot _y _stderr _t1 _t2 id, tau2(loop)

**Cognitive flexibility**

#1 network setup mean sd n, study(id) trt(t) format(augment) smd

#2 network map, improve

#3 network meta i

#4 network meta c

#5 network forest

#6 network sidesplit all, tau

#7 clear

#8 network setup mean sd n, study(id) trt(t) format(augment) smd

#9 network meta i

#10 network meta c

#11 network rank min, all zero reps(5000) gen(prob)

#12 sucra prob*, lab(AE RE MBE TAU)

#13 netleague, lab(AE RE MBE TAU) sort(AE MBE RE TAU)

#14 intervalplot, null(0) lab(AE RE MBE TAU)

#15 network convert pairs

#16 netfunnel _y _stderr _t1 _t2 , random bycomp add(lfit _stderr _ES_CEN) noalpha

#17 ifplot _y _stderr _t1 _t2 id, tau2(loop)
